# Detecting Bacteria in Their Mammalian Hosts Using Metabolism-Targeted [^13^C]CO_2_ Breath Testing

**DOI:** 10.1021/acscentsci.5c01995

**Published:** 2026-03-18

**Authors:** Marina López-Álvarez, Sang Hee Lee, Anju Wadhwa, Mohammad Yaqoob Bhat, Tyler S. Simmons, Jung Min Kim, Anil P. Bidkar, Spenser R. Simpson, Shari Dhaene, Jeffrey D. Steinberg, Joseph Blecha, Robert R. Flavell, Marshall D. McCue, Amanda M. Green, Renuka Sriram, Tom Desmet, Joanne Engel, Jason W. Rosch, Michael A. Ohliger, Kiel D. Neumann, David M. Wilson

**Affiliations:** † Department of Radiology and Biomedical Imaging, 8785 University of California, San Francisco, San Francisco, California 94158, United States; ‡ Department of Host-Microbe Interactions, St. Jude Children’s Research Hospital, Memphis, Tennessee 38105, United States; § Department of Radiology, St. Jude Children’s Research Hospital, Memphis, Tennessee 38105, United States; ∥ Department of Biotechnology, Centre for Synthetic Biology, Ghent University, Gent B-9000, Belgium; ⊥ Center for In Vivo Imaging and Therapeutics, St. Jude Children’s Research Hospital, Memphis, Tennessee 38105, United States; # 525875 Sable Systems International, Las Vegas, Nevada 89032, United States; ∇ Department of Infectious Diseases, St. Jude Children’s Research Hospital, Memphis, Tennessee 38105, United States; ○ Department of Medicine, University of California, San Francisco, San Francisco, California 94158, United States; ◆ Department of Radiology, Zuckerberg San Francisco General Hospital, San Francisco, California 94110, United States; ¶ Center for Infectious Disease Research, St. Jude Children’s Research Hospital, Memphis, Tennessee 38105, United States

## Abstract

Infectious diseases are a major cause of morbidity and mortality worldwide. With the increasing frequency of antibiotic resistance, efficient and noninvasive diagnostic methods are more important than ever. In this report, we interrogate the use of several intravenously administered, bacteria-specific, ^13^C-enriched metabolites whose conversion to [^13^C]­CO_2_ can be detected via a portable and inexpensive method, namely nondispersive infrared (NDIR) spectroscopy. The enriched metabolites [U-^13^C]­maltose, [U-^13^C]­maltotriose, d-[U-^13^C]­mannitol, and l-[U-^13^C]­arabinose were metabolized to [^13^C]­CO_2_ by several pathogens *in vitro*, while showing minimal [^13^C]­CO_2_ production in uninfected mice. We further demonstrated that myositis, bacteremia, pneumonia, and osteomyelitis could be detected *in vivo* using one or more ^13^C-enriched metabolites. Additionally, in a model of *Escherichia coli* myositis, [^13^C]­CO_2_ production correlated with bacterial burden following ceftriaxone therapy, showing that exhaled [^13^C]­CO_2_ could be employed to monitor antimicrobial efficacy. Finally, [^13^C]­CO_2_ production by *Staphylococcus aureus* clinical isolates treated with [U-^13^C]­maltose was correlated with the performance of its cognate PET tracer [2-^18^F]­maltose, suggesting that [^13^C]­CO_2_ breath testing could predict the performance of pathogen-targeted positron emission tomography (PET) tracers *in vivo*. [^13^C]­CO_2_ breath testing using an expanded metabolite toolbox and on-site detection tools represents a unique and complementary method to identify bacterial infection in clinical practice.

## Graphical abstract

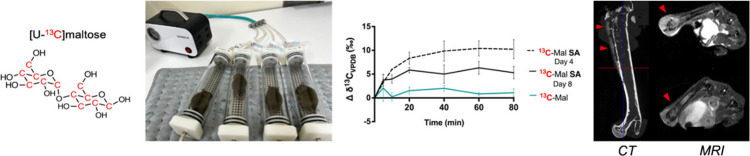

## Introduction

Accurately and quickly distinguishing invasive bacterial infection from viral illnesses and noninfectious conditions remains a critical challenge in clinical medicine, accelerating the development of diagnostic methods targeting pathogen-specific metabolism. These techniques may be especially useful in the acute setting when polymerase chain reaction (PCR)-based methods^1^ and routine culture/sensitivity results are not immediately available. Recent efforts have focused on new infection imaging methods for positron emission tomography (PET),^2,3^ or magnetic resonance imaging (MRI)^4^ to better identify active infection especially via direct targeting of bacteria themselves rather than the host immune response. Elegant *in silico* and *in vitro* analyses of bacterial metabolism^5^ have motivated the radiolabeling of sugars and sugar alcohols that are efficiently metabolized by bacteria but not humans, such as 2-deoxy-[2-^18^F]­fluorosorbitol ([^18^F]­FDS), [2/6-^18^F]­fluoromaltose, 6”-[^18^F]­fluoromaltotriose, and d-[2-^18^F]­mannitol.^6–8^ When combined with PET/computed tomography (PET/CT) or PET/magnetic resonance (PET/MR), these tracers may be useful not only in detecting the presence of infection but also in identifying its location and response to antimicrobial therapy. Based on compelling preclinical data, demonstrating utility in humans with infections is the next step- ideally comparing several promising methods in clinical trials.

Motivated by these recent discoveries, we sought analogous noninvasive methods to detect living bacteria within their mammalian hosts noninvasively (Figure 1). The application of metabolism-targeted [^13^C]­CO_2_ breath testing was inspired by the clinical use of [^13^C]­urea in microbial identification. Several microorganisms including *Helicobacter pylori*, *Staphylococcus aureus, Staphylococcus epidermidis*, and *Klebsiella pneumoniae* produce urease (E.C. 3.5.1.5), a cytosolic enzyme that catalyzes the hydrolysis of urea to ammonia and carbon dioxide. Urease production is the basis for diagnosing *H. pylori*-induced gastritis, gastric ulcer, and peptic ulcer disease via [^13^C]­CO_2_ breath testing.^9,10^ A urea breath test study is almost exclusively performed in the outpatient setting, whereby [^13^C]­urea is administered *orally* and after 10–30 min liberated [^13^C]­CO_2_ in the exhaled breath is analyzed via isotope ratio mass spectroscopy (IRMS)^11^ with Δδ^13^C_VPDB_(‰) > 5 typically indicating a positive test.^12^ IRMS typically requires sending the sample to a specialized facility as many academic institutions do not have on-site IRMS systems, while nondispersive infrared (NDIR) spectroscopy^13,14^ or integrated cavity output spectroscopy (ICOS)^15,16^ can be performed using a table-top instrument (Table S1). Beyond the use of [^13^C]­CO_2_ in *H. pylori* detection, breath testing has been used in diverse settings, including testing carbohydrate assimilation via [^13^C]­lactose,^17^ exocrine pancreatic function via [^13^C]­triglycerides,^18,19^ and liver function via [^13^C]­phenylalanine.^20^ To the best of our knowledge, other than [^13^C]­urea, the only carbon-13 enriched metabolites used to detect bacteria in patients are d-[^13^C]­xylose^21^ and [^13^C]­glycocholate, which are administered orally to identify pathologic activity of gastrointestinal flora associated with chronic and inflammatory bowel diseases, known as small intestine bacterial overgrowth (SIBO).^22^ We sought to identify invasive bacterial infections, distinguishing them from colonizing bacteria in the acute care setting. Imaging data suggest that commensal bacteria do not significantly metabolize intravascularly delivered substrates, whereas bacteria in infected tissue do.^8,23,24^ We hypothesized that intravenous administration of several commercially available ^13^C-enriched metabolites could be used to detect bacterial infections in their mammalian hosts via exhaled [^13^C]­CO_2,_ detected by NDIR or integrated cavity output spectroscopy (ICOS). These metabolites include urea, l-arabinose, d-sorbitol, d-xylose, maltose, maltotriose, and d-mannitol, substrates not readily metabolized by mammals. A prerequisite for their clinical application would be minimal or absent [^13^C]­CO_2_ production by uninfected patients.

**Figure 1:**
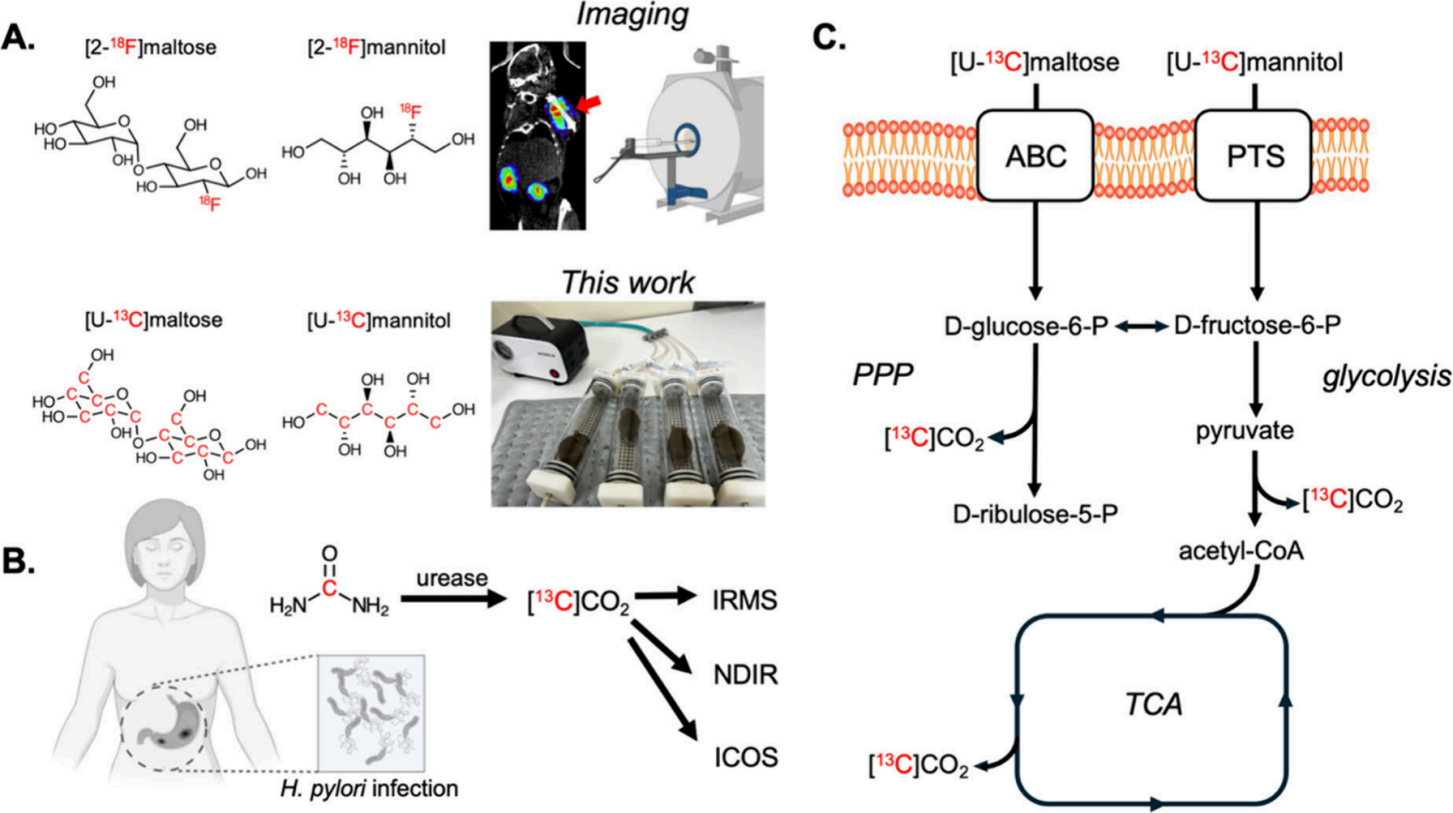
Rationale for using bacteria-specific metabolic pathways in conjunction with [^13^C]­CO_**2**_ breath testing to detect infection in patients. **A**. Numerous recently developed positron emission tomography (PET) radiotracers target bacteria-specific metabolic pathways, including fluorine-18 labeled [2-^18^F]­maltose and d-[2-^18^F]­mannitol. These can readily distinguish acute bacterial infection from sterile inflammation using μPET-CT. These imaging data motivated the development of ^13^C-enriched metabolites for [^13^C]­CO_2_ breath testing including the [U-^13^C]­maltose and d-[U-^13^C]­mannitol homologues shown. Infected mice were studied in a metabolic chamber following *intravenous* metabolite administration with their [^13^C]­CO_2_ production followed using nondispersive infrared (NDIR) spectroscopy. **B**. Breath testing for *H. pylori* in the clinic uses *orally* administered [^13^C]­urea that is metabolized in the stomach/duodenum via urease (EC 3.5.1.5) into [^13^C]­CO_2_ that may be detected via isotope ratio mass spectroscopy (IRMS), NDIR spectroscopy or integrated cavity output spectroscopy (ICOS). **C**. Metabolism of maltose and d-mannitol by *S. aureus* illustrating several potential sources of [^13^C]­CO_2_ production. Following transport, maltose is hydrolyzed to d-glucose catalyzed by α-1,4-glucosidase (EC 3.2.1.20) yielding d-fructose-6-P after phosphorylation/isomerization. d-fructose-6-P is also produced by the transport and phosphorylation/isomerization of d-mannitol. Downstream [^13^C]­CO_2_ is produced by the decarboxylation of pyruvate, isocitrate and α-ketoglutarate.

In this manuscript, we expand the scope of breath testing for infection to include numerous ^13^C-enriched metabolites that are administered intravenously rather than orally, with [^13^C]­CO_2_ production detected via NDIR. The universally ^13^C-enriched metabolites [U-^13^C]­maltose, d-[U-^13^C]­mannitol, and l-[U-^13^C]­arabinose showed little or no [^13^C]­CO_2_ signals in uninfected animals but robust [^13^C]­CO_2_ production in models of bacterial infection, indicating that invasive bacterial infections could be quickly and noninvasively identified within their mammalian hosts. Taken together, these data support [^13^C]­CO_2_ breath testing as a powerful tool to complement existing diagnostic methods in clinical practice.

## Results

### *In Vitro* Screening of Universally ^13^C-Enriched Metabolites Showed [^13^C]­CO_2_ Production for Several Substrate/Pathogen Combinations

To predict the utility of [^13^C]­CO_2_ breath testing to detect bacterial infections *in vivo*, we first determined using NDIR whether commercially available, universally ^13^C-enriched and potentially bacteria-specific substrates were metabolized to [^13^C]­CO_2_ in log-phase bacterial cultures. This screening method represents an efficient alternative to ^3^H/^14^C labeled metabolite/scintillation counting,^5,25^ nuclear magnetic resonance (NMR), or mass spectrometry for identifying bacterial incorporation of exogenous molecules. Production of [^13^C]­CO_2_ after the incubation of ^13^C-labeled compounds with human pathogenic Gram-positive (*Staphylococcus aureus*, *S. epidermidis*, *Enterococcus faecalis*), and Gram-negative (*Escherichia coli*, *Salmonella typhimurium*, *Enterobacter cloacae*) bacteria (Table S2) was studied (Figure 2, Table S3). We used d-[U-^13^C]­glucose as a positive control and [^13^C]­urea as a negative control in nonurease producing organisms (including the *S. aureus* strain studied). As expected, all bacteria produced high [^13^C]­CO_2_ when incubated with d-[U-^13^C]­glucose, whereas minimal [^13^C]­CO_2_ production was seen using [^13^C]­urea, except for in *S. epidermidis* cultures. Despite reports describing d-xylose metabolism in *E. coli*,^26^ and the reported application of xylose to breath testing for SIBO,^27^ no [^13^C]­CO_2_ was detected upon incubation of *E. coli* with d-[U-^13^C]­xylose. Several bacteria produced [^13^C]­CO_2_ when incubated with [U-^13^C]­maltose, [U-^13^C]­maltotriose, d-[U-^13^C]­mannitol, l-[U-^13^C]­arabinose and d-[U-^13^C]­sorbitol, while no [^13^C]­CO_2_ production was observed after incubation with their naturally abundant correlates (Supp. Figure S1).

**Figure 2:**
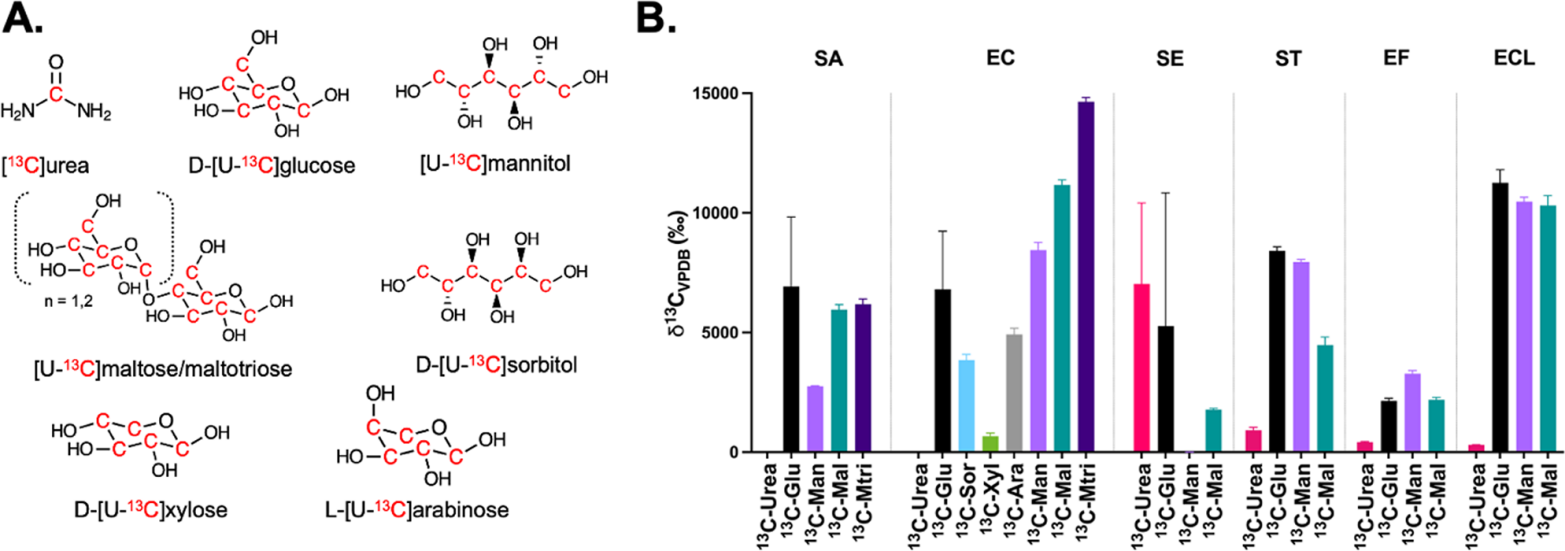
*In vitro* production of [^13^C]­CO_2_ by human pathogenic bacteria. **A**. Chemical structures of commercially available universally ^13^C-enriched metabolites. **B**. Production of [^13^C]­CO_2_ *in vitro* was studied in *S. aureus* (SA), *E. coli* (EC), *S. epidermidis* (SE), *S. typhimurium* (ST), *E. faecalis* (EF) and *E. cloacae* (ECL) cultures. [^13^C]­urea served as a negative control for nonurease producing bacteria (EC, ST, EF, ECL) while d-[U-^13^C]­glucose (^13^C-Glu) served as a positive control for all the bacteria tested. All cultures demonstrated robust production of [^13^C]­CO_2_ using d-[U-^13^C]­glucose while only *S. epidermidis* (urease-producing) showed significant [^13^C]­CO_2_ production following [^13^C]­urea administration. Several bacteria produced [^13^C]­CO_2_ when incubated with [U-^13^C]­maltose (^13^C-Mal; SA, EC, SE, ST, EF, ECL), [U-^13^C]­maltotriose (^13^C-Mtri; SA, EC), d-[U-^13^C]­mannitol (^13^C-Man; SA, EC, ST, EF, ECL), l-[U-^13^C]­arabinose (^13^C-Ara; EC) and d-[U-^13^C]­sorbitol (^13^C-Sor; EC).

To investigate the minimum amount of ^13^C-enriched metabolite that was necessary to detect [^13^C]­CO_2_ production, identical quantities of *S. aureus* and *E. coli* were incubated with decreasing metabolite concentrations. For *S. aureus*, [^13^C]­CO_2_ production could be detected at concentrations as low as 25 μM for d-[U-^13^C]­mannitol, d-[U-^13^C]­glucose, [U-^13^C]­maltose, and [U-^13^C]­maltotriose (Supp. Figures S2–S3). For *E. coli*, [^13^C]­CO_2_ production could be detected at concentrations as low as 25 μM for d-[U-^13^C]­mannitol, d-[U-^13^C]­glucose, [U-^13^C]­maltose, and [U-^13^C]­maltotriose, and as low as 50 mM for l-[U-^13^C]­arabinose and d-[U-^13^C]­sorbitol, but not below 1000 μM for d-[U-^13^C]­xylose.

Finally, we compared [^13^C]­CO_2_ detection techniques. *S. aureus* cultures were treated with d-[U-^13^C]­glucose as above, and [^13^C]­CO_2_ detection was found to be similar using IRMS and NDIR (Supp. Figure S4). These results suggested that ^13^C-enriched metabolites could be useful for detecting important Gram-positive and Gram-negative human bacterial pathogens *in vivo* via [^13^C]­CO_2_ breath testing in conjunction with NDIR.

### Administration of Candidate ^13^C-Enriched Metabolites to Uninfected Mice Resulted in Minimal [^13^C]­CO_2_ Exhalation

To further evaluate ^13^C-enriched metabolites for detecting bacteria *in vivo*, we screened commercially available molecules in uninfected animals to select those with low background levels of [^13^C]­CO_2_ production (Figure 3A). Due to the presence of commensal bacteria in the gastrointestinal tract, we hypothesized that intravenous administration of ^13^C-enriched metabolites was critical to detect many infections. When d-[U-^13^C]­mannitol was administered orally to healthy mice, significant [^13^C]­CO_2_ production was observed, consistent with the presence of *E. coli* in the murine microbiome (Supp. Figure S5). For all other studies in uninfected mice, metabolites were injected intravenously, followed by gas sampling in a metabolic chamber for 100 min and subsequent NDIR analysis. d-[U-^13^C]­glucose and d-[U-^13^C]­sorbitol resulted in robust [^13^C]­CO_2_ production, as expected given that they are metabolized by mammalian cells. Observable [^13^C]­CO_2_ was also produced after intravenous administration of d-[U-^13^C]­xylose and [^13^C]­urea, which were not further evaluated as bacteria-specific breath testing agents. In contrast, injection of [U-^13^C]­maltose, [U-^13^C]­maltotriose, d-[U-^13^C]­mannitol, and l-[U-^13^C]­arabinose resulted in minimal [^13^C]­CO_2_ production, demonstrating their potential utility for detecting pathogens *in vivo.* Based on these *in vitro* and *in vivo* studies, and the prohibitively high cost of [U-^13^C]­maltotriose, subsequent studies in infected animals were conducted with [U-^13^C]­maltose, d-[U-^13^C]­mannitol, and l-[U-^13^C]­arabinose.

**Figure 3:**
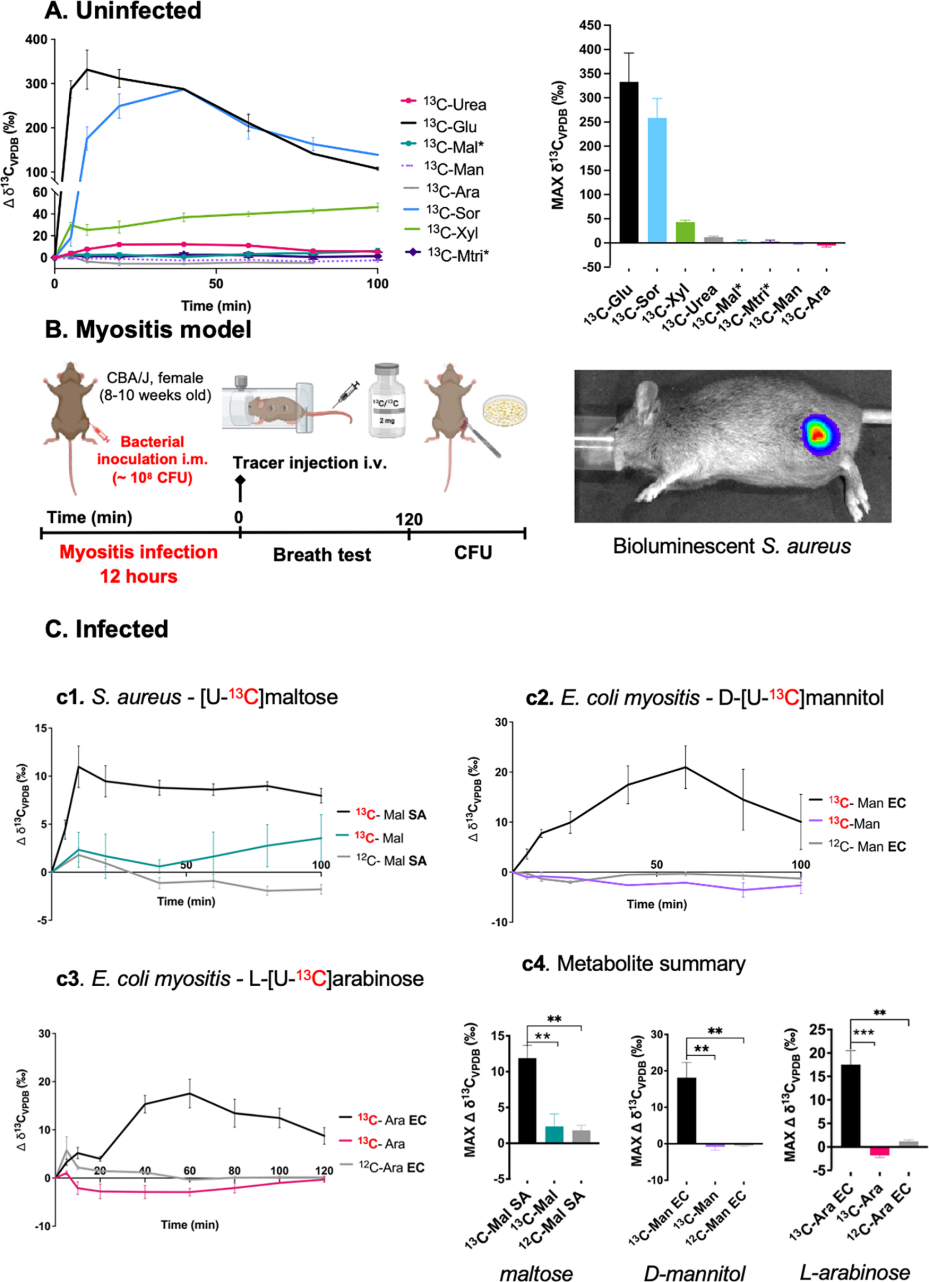
*In vivo* **[** ^13^C]­CO_2_ production in both uninfected and infected animals following administration of universally ^13^C-enriched metabolites. **A**. In normal animals, both intravenous d-[^13^C]­glucose (^13^C-Glu) and d-[U-^13^C]­sorbitol (^13^C-Sor) resulted in large [^13^C]­CO_2_ signals in ∼10 min with d-[U-^13^C]­xylose (^13^C-Xyl) and [^13^C]­urea also showing subtle elevated signals. In contrast, [U-^13^C]­maltose* (^13^C-Mal), [U-^13^C]­maltotriose* (^13^C-Mtri), d-[U-^13^C]­mannitol (^13^C-Man), and l-[U-^13^C]­arabinose (^13^C-Ara) showed little or no [^13^C]­CO_2_ production. **B**. Schematic of murine myositis model. A murine myositis model was first studied to detect bacteria-specific metabolite conversion. In all cases the cohorts were comprised by *N* = 4 animals per group, with 2 mg of universally enriched ^13^C metabolite administered intravenously per animal. To promote the serum stability expected in humans, *­[U-^13^C]­maltose and [U-^13^C]­maltotriose were simultaneously administered with voglibose (1 mg per animal). Bioluminescence imaging (BLI) confirmed the successful development of myositis. **C**. Exhaled [^13^C]­CO_2_ by infected mice following ^13^C-enriched metabolite administration. **c1**. In *S. aureus* (SA)-infected mice studied with [U-^13^C]­maltose*, increased [^13^C]­CO_2_ production was observed from all the infected mice; bacterial burden was *∼*10^11^ CFUs. In contrast elevated signals were not observed for either uninfected mice (**P-value = 0.0068) or infected mice intravenously injected with natural abundance maltose (^12^C-Mal; **P-value = 0.0078). **c2**. In *E. coli* (EC)-infected mice injected with d-[U-^13^C]­mannitol, elevated [^13^C]­CO_2_ signals were seen but not in the corresponding uninfected mice (**P-value= 0.0043) or in mice intravenously injected with natural abundance mannitol (^12^C-Man; **P-value = 0.0043). **c3**. In *E. coli*-infected mice studied with l-[U-^13^C]­arabinose, elevated [^13^C]­CO_2_ signals were seen in contrast to the corresponding uninfected mice (***P-value= 0.0007) or mice intravenously injected with natural abundance arabinose (^12^C-Ara;**P-value= 0.0015). P-values were determined by two-tailed Student’s *t* test (Graphpad Prism 10) of the maximum Δ δ^13^C_VPDB_ (‰) time value. **c4**. Summary of maximum Δ δ^13^C_VPDB_ (‰) in infected animals versus uninfected and natural abundance controls.

### Administration of [U-^13^C]­Maltose, d-[U-^13^C]­Mannitol, and l-[U-^13^C]­Arabinose Resulted in Increased Production of [^13^C]­CO_2_ in Infected Mice That Decreased Following Antibiotic Therapy

After showing low background signals in uninfected mice for these metabolites, we tested the diagnostic capability of these metabolites *in vivo* using a well-established murine myositis model (Figure 3A).^6,23^ In *S. aureus*-infected mice, significantly increased [^13^C]­CO_2_ production was observed immediately following [U-^13^C]­maltose administration, in contrast to uninfected mice or infected animals that were injected with natural abundance (^12^C) maltose (Figure 3c1). Significantly increased [^13^C]­CO_2_ production was also observed in *E. coli*-infected mice after administration of d-[U-^13^C]­mannitol and l-[U-^13^C]­arabinose, in contrast to uninfected mice or natural abundance controls (Figure 3c2, c3). The effect of host fasting on [^13^C]­CO_2_ production was also evaluated in *E. coli-*infected mice after administration of d-[U-^13^C]­mannitol (Supp. Figure S6). [^13^C]­CO_2_ signals were similar in the fed and fasted states, but potential differences should be evaluated explicitly in future human studies.

In clinical practice, a major failure of diagnostic imaging techniques (plain-film, CT and MR) is their inability to determine whether infection is responding appropriately to antibiotic therapy in a timely manner, as radiologic improvements usually lag behind microbiological and clinical improvement. Thus, it would be of great benefit to use bacteria-specific metabolites to detect changes in bacterial load in real time and thus optimize antimicrobial therapy. To demonstrate that antibiotic treatment efficacy could be detected via [^13^C]­CO_2_ breath testing using d-[U-^13^C]­mannitol, mice with induced *E. coli* myositis were treated with 5 mg/kg ceftriaxone subcutaneously every 90 min for 24 h, followed by observation in a metabolic chamber for 100 min (Figure 4A). [^13^C]­CO_2_ signals prior to ceftriaxone therapy were compared to those following therapy in the same murine cohort. Infected mice treated with ceftriaxone showed decreased [^13^C]­CO_2_ production following therapy, consistent with a reduced infection burden (∼10^5^ CFUs versus ∼10^10^ CFUs prior to treatment), as determined by tissue homogenization and plating (Figure 4B).

**Figure 4:**
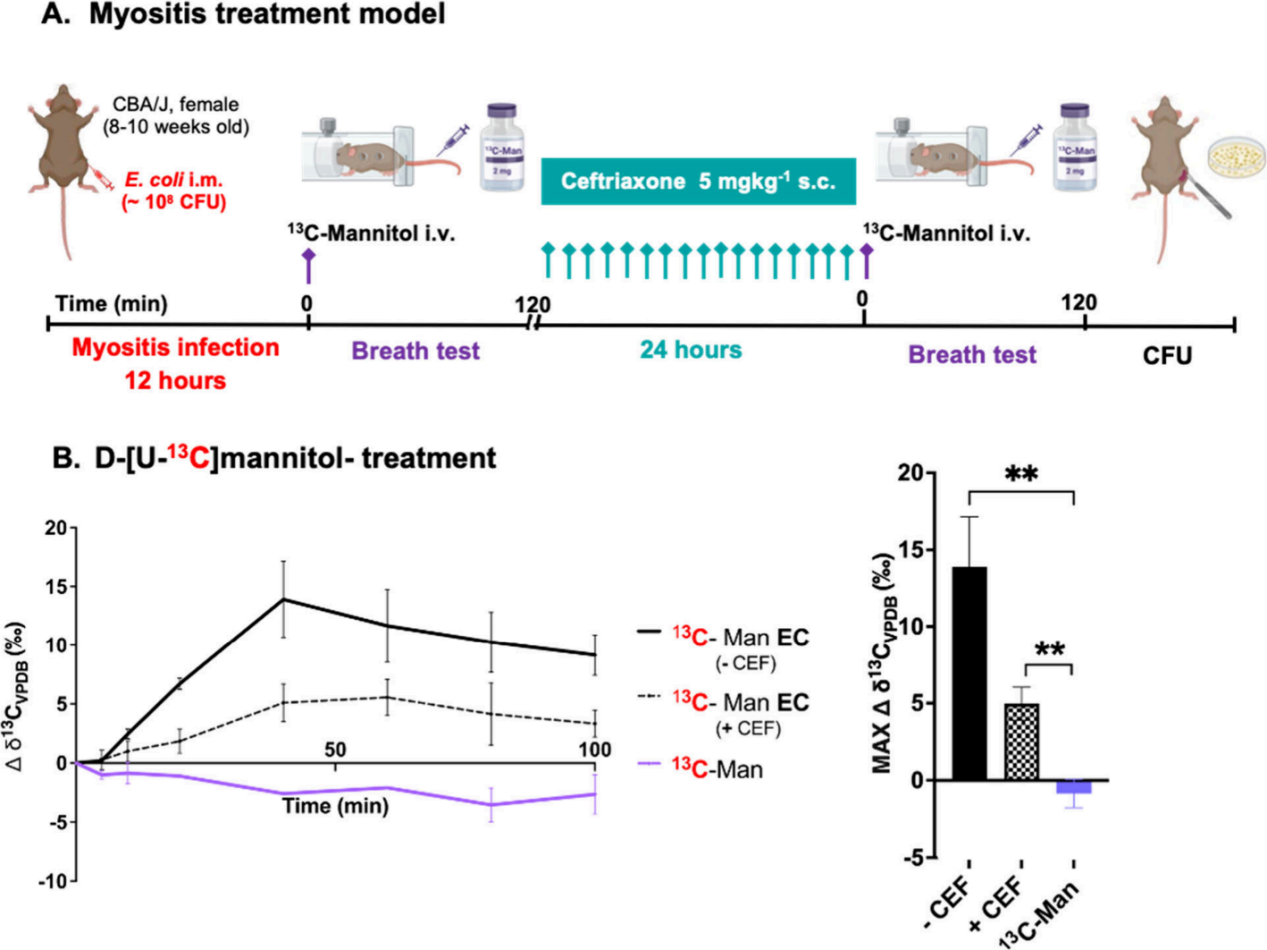
*In vivo* [^13^C]­CO_2_ production in mice infected with *E. coli* prior and after antibiotic treatment. **A**. *E. coli* (EC) was inoculated into the right hind leg muscle, followed by IV administration of d-[U-^13^C]­mannitol at 12 h post inoculation. Following breath test evaluation, mice were treated with ceftriaxone (CEF) every 90 min for 24 h. The same mice were then studied with a second breath test. P-values were determined by two-tailed Student’s *t* test (Graphpad Prism 10) of the maximum Δ δ^13^C_VPDB_ (‰) time value. *N* = 4 per group. **B**. *In vivo* [^13^C]­CO_2_ production from d-[U-^13^C]­mannitol (^13^C-Man) in mice infected with *E. coli* (EC) prior to treatment (-CEF; ∼10^10^ CFU, **P-value = 0.0046) and after treatment (+CEF; ∼10^5^ CFU, **P-value = 0.0090).

### Breath Tests with d-[U-^13^C]­Mannitol and [U-^13^C]­Maltose Identified Infection in Preclinical Models of Bacteremia, Pneumonia, and Osteomyelitis

To explore the breadth of potential clinical uses for [^13^C]­CO_2_ breath testing we evaluated murine infection models of bacteremia, pneumonia, and osteomyelitis. Based on results in the myositis model, *E. coli* bacteremia was studied using d-[U-^13^C]­mannitol while preclinical models of *S. aureus* pneumonia and osteomyelitis were evaluated using [U-^13^C]­maltose. For the bacteremia model, intraperitoneal inoculation of 10^6^ CFUs *E. coli* ^28^ was followed by d-[U-^13^C]­mannitol injection at 12 h and subsequent [^13^C]­CO_2_ monitoring (Figure 5A). Following [^13^C]­CO_2_ testing, select tissues were homogenized, serially diluted and plated for CFU counting; the highest bacterial burden was observed in the liver, followed by spleen and kidneys. Elevated [^13^C]­CO_2_ production was observed for *E. coli*-infected mice but not for uninfected mice, nor for infected mice injected with natural abundance d-mannitol (Figure 5B). For the *S. aureus* pneumonia model, mice were intranasally inoculated with 10^7^ CFUs, injected with d-[U-^13^C]­maltose at 24 h post-inoculation, and monitored for [^13^C]­CO_2_ production^29^ (Figure 6A). Increased [^13^C]­CO_2_ production was observed for *S. aureus*-infected mice but not for uninfected mice, mice inoculated with heat-killed bacteria, or infected mice injected with natural abundance maltose (Figure 6B). For the osteomyelitis model, *S. aureus* (10^7^ CFUs) was directly inoculated intrafemorally into the right femur. At either 4- or 8-days post injection, [U-^13^C] maltose or the natural abundance metabolite was intravenously injected, and subsequent [^13^C]­CO_2_ production was monitored (Figure 7A). Mice injected with [U-^13^C]­maltose showed elevated [^13^C]­CO_2_ production relative to uninfected and natural abundance controls at both time points (Figure 7B). For cohorts studied at 8 days post-inoculation, [^13^C]­CO_2_ production reflected bacterial burden as quantified by *ex vivo* tissue homogenization and plating. Mice whose femoral bacterial burden was ∼ 10^7^ CFUs had higher [^13^C]­CO_2_ production than mice whose femoral bacterial burden was ∼10^5^ CFUs, with all infected mice yielding more [^13^C]­CO_2_ than uninfected or natural abundance controls (Figure 7C, Supp. Figure S12). Correlative imaging studies performed at days 7 and 8 showed characteristic abnormalities of osteomyelitis in the inoculated femur (Figure 7D). μPET-CT using [2-^18^F]­maltose on day 7 showed elevated femoral uptake, highlighting the potential synergy between [U-^13^C]­maltose breath testing and PET imaging. MRI on day 7 showed abnormal signals within bone and adjacent soft tissues, and high-resolution CT following animal sacrifice on day 8 demonstrated cortical destruction and periosteal reaction.

**Figure 5:**
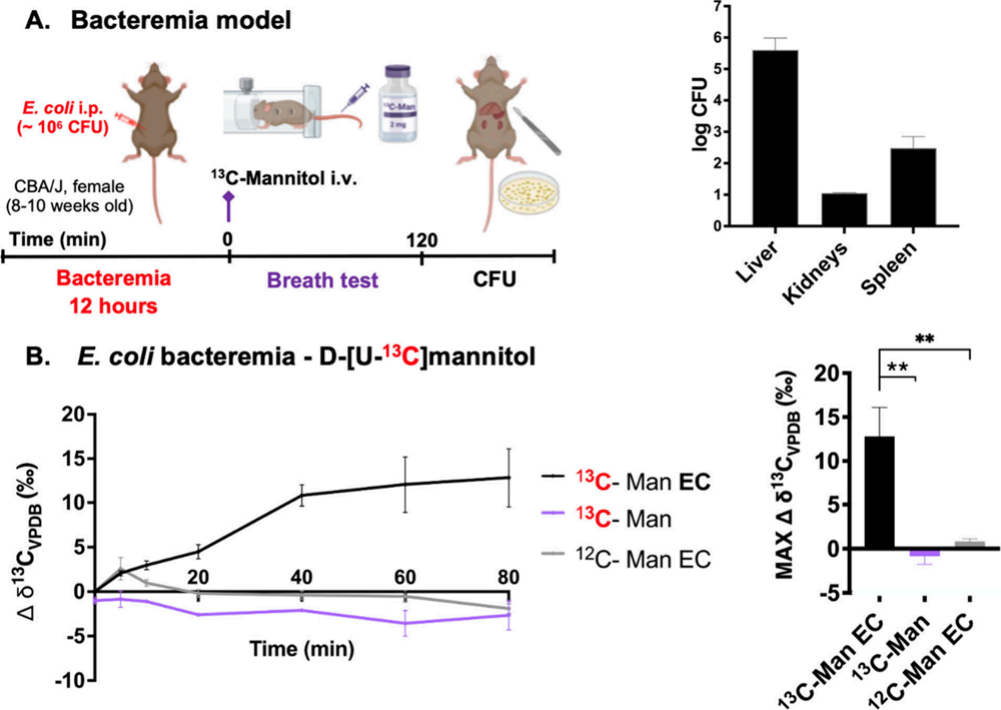
*In vivo* **[** ^13^C]­CO_2_ production in mice with *E. coli* bacteremia and healthy mice following administration of d-[U-^13^C]­mannitol. **A**. Bacteremia model scheme and colony forming units (CFU) of Liver, Kidneys and spleen to verify the successful establishment of the infection. P-values were determined by two-tailed Student’s *t* test (Graphpad Prism 10) of the maximum Δδ^13^C_VPDB_ (‰) time value. *N* = 4 per group. **B**. In *E. coli* (EC) bacteremia mice studied with d-[U-^13^C]­mannitol (^13^C-Man) increased [^13^C]­CO_2_ production was observed from all the infected animals. In contrast elevated signals were not observed for either uninfected mice (**P-value = 0.0051) or infected animals studied using an identical dose of natural abundance mannitol (^12^C-Man) (**P-value = 0.0044).

**Figure 6:**
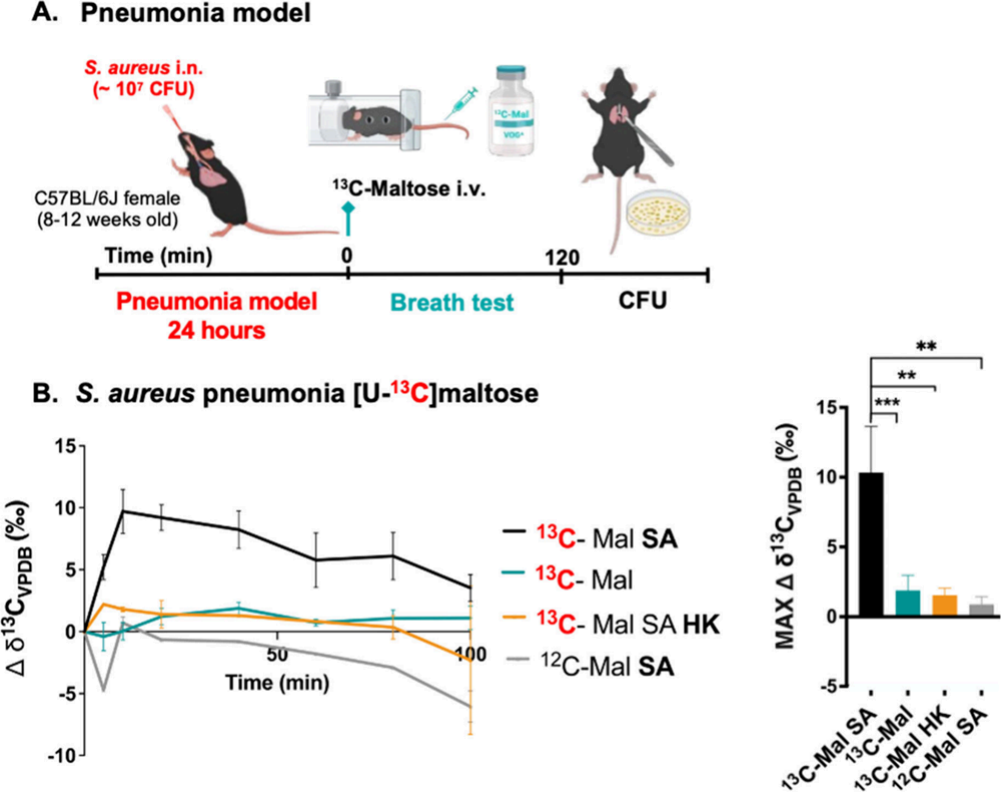
*In vivo* **[** ^13^C]­CO_2_ production by *S. aureus* in a murine lung infection model (*N* = 16) following intravenous [U-^13^C]­maltose injection. **A**. Lung infection model timeline. **B**. In infected mice (SA) studied with [U-^13^C]­maltose (^13^C-Mal) increased [^13^C]­CO_2_ production was observed from all the infected animals. In contrast elevated signals were not observed for either uninfected mice (***P-value = 0.0001), infected animals studied using an identical dose of natural abundance maltose (^12^C- Mal; **P-value = 0.0050) or animals inoculated with heat-killed *S. aureus* (SA HK) (**P-value = 0.0067). *P*-values were determined by two-tailed Student’s *t* test (Graphpad Prism 10) of the maximum Δ δ^13^C_VPDB_ (‰) time value. *N* = 4 per group.

**Figure 7:**
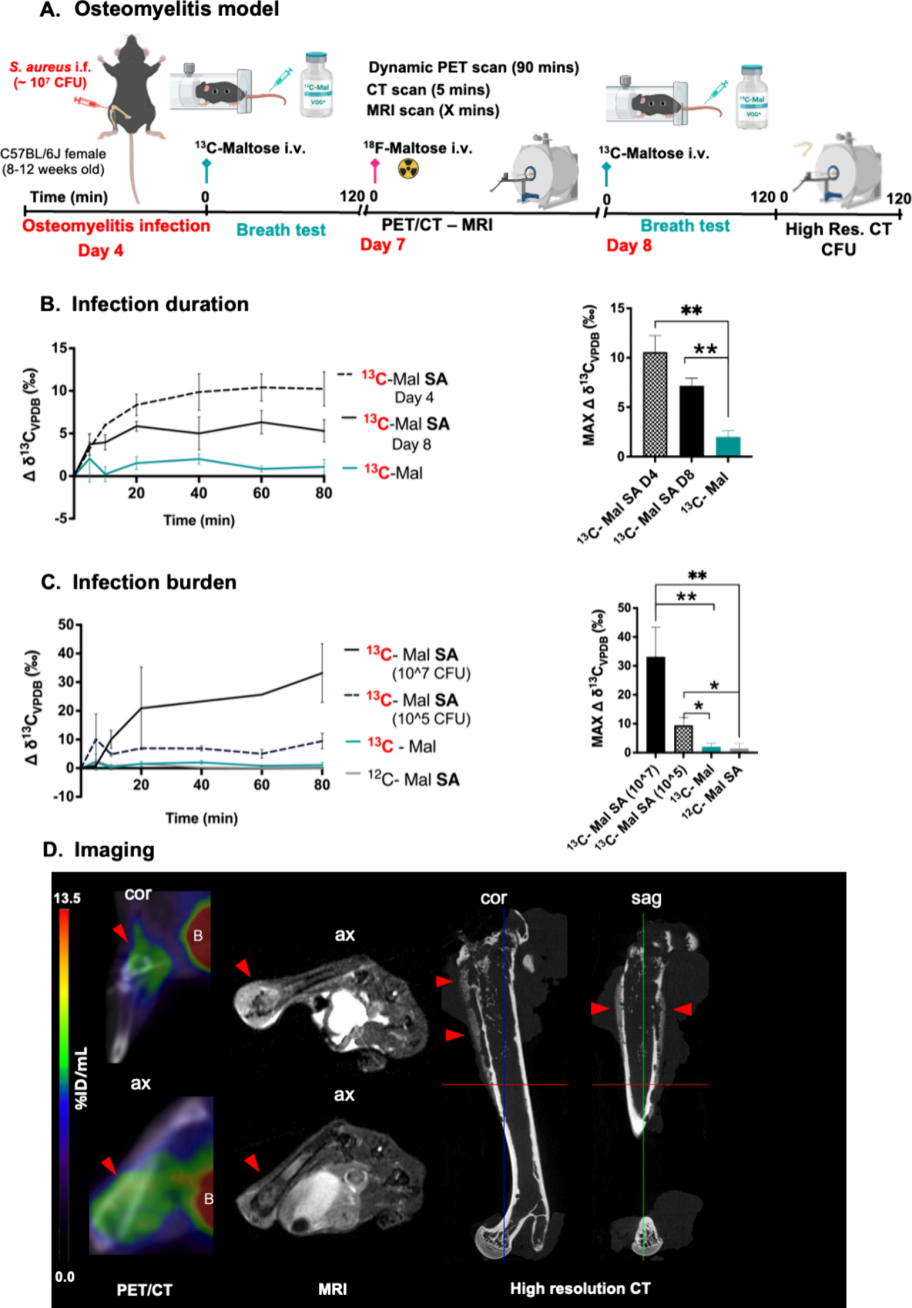
*In vivo* **[** ^13^C]­CO_2_ production by *S. aureus* in a murine osteomyelitis model studied using [U-^13^C]­maltose. **A**. Osteomyelitis infection model timeline. **B**. [^13^C]­CO_2_ production by animals infected with *S. aureus* (SA) after administration of [U-^13^C]­maltose (^13^C-Mal) (*N* = 11). Animals infected with *S. aureus* showed increased [^13^C]­CO_2_ production at an earlier time point following infection, with greater signals seen at 4 days (**P-value = 0.0029) than at 8 days (**P-value = 0.0020). **C**. [^13^C]­CO_2_ production by animals infected with a higher CFU concentration (∼10^7^, *N* = 3) was higher than that seen for a lower CFU concentration (∼10^5^, *N* = 3). All infected animals showed increased [^13^C]­CO_2_ production compared to healthy animals (**P-value = 0.0080, *P-value = 0.0243, *N* = 4) and ^12^C-maltose (^12^C-Mal) controls (**P-value = 0.0078, *P-value = 0.0243, *N* = 4). *P*-values were determined by two-tailed Student’s *t* test (Graphpad Prism 10) of the maximum Δ δ^13^C_VPDB_ (‰) time value. **D**. Correlative imaging data using μPET/CT ([2-^18^F]­maltose), MRI and high-resolution CT. The sites of infection are indicated by red arrows. Axial (ax), coronal (cor), sagittal (sag).

### [^13^C]­CO_2_ Production by MRSA Cultures Treated with [U-^13^C]­Maltose Was Correlated with *in Vitro* [2-^18^F]­Maltose Accumulation, and *in Vivo* [2-^18^F]­Maltose Signals in Infected Mice

One significant challenge in bacteria-specific PET imaging is that different bacterial species and even strains demonstrate variable radiotracer uptake. This variability could potentially lead to false-negative exams for infections caused by bacteria that are insensitive to a given PET tracer. Breath testing using a ^13^C-enriched metabolite might therefore be applied as a screening tool to predict the behavior of a structurally and biochemically analogous imaging probe. To test this hypothesis, we compared the uptake of [2-^18^F]­maltose and [U-^13^C]­maltose in four different methicillin resistant *S. aureus* (MRSA) clinical isolates. All four isolates grew at a similar rate *in vitro* (Supp. Figure S13A). The accumulation of [2-^18^F]­maltose and the production of [^13^C]­CO_2_ using [U-^13^C]­maltose showed similar trends in all four strains: high for 2 strains and low for 2 strains (Figure 8 A,B). We subsequently studied one “high” uptake and one “low” uptake strain in the same murine model of myositis^24^ using μPET-CT. The bacterial burdens for both strains were nearly identical as verified by serial dilution and plating (Supp. Figure S13B). However, the difference in PET signals was visibly apparent (Figure 8C), with *ex vivo* biodistribution data confirming higher [2-^18^F]­maltose accumulation in tissues infected with the higher [^13^C]­CO_2_-producing strain (Figure 8D). This correlation between [^13^C]­CO_2_ breath testing and μPET-CT might be exploited in clinical scenarios for which identification of bacterial infection is followed by its precise localization.

**Figure 8:**
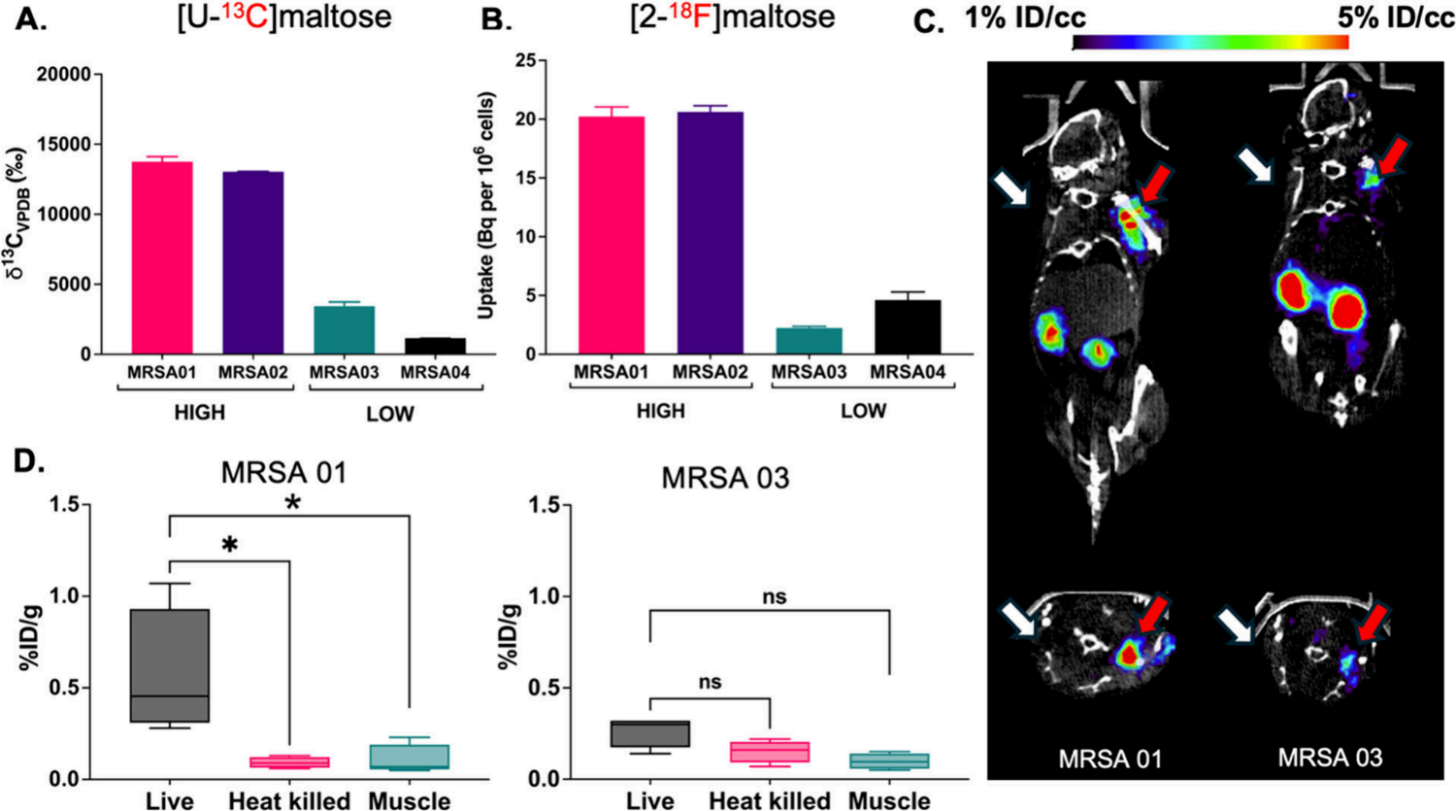
Correlation between *in vitro* [^13^C]­CO_2_ production, *in vitro* [2**-** ^18^F]­maltose uptake, and *in vivo* [2**-** ^18^F]­maltose signals. Four clinical strains of methicillin-resistant *S. aureus* (MRSA) were identified, two of which had higher uptake of [2-^18^F]­maltose (MRSA 01 and MRSA 02) and two of which had lower uptake of [2-^18^F]­maltose (MRSA 03 and MRSA 04). This heterogeneity among strains represents a challenge in pathogen-specific imaging. **A, B**. *In vitro* [^13^C]­CO_2_ production following [U-^13^C]­maltose administration and [2-^18^F]­maltose accumulation show a similar pattern between the strains. **C**. μPET/CT imaging of MRSA 01 and MRSA 03 using [2-^18^F]­maltose. For both MRSA 01 (high uptake) and MRSA 03 (low uptake) the site of live inoculation is indicated by a red arrow and the site of 10× heat-killed inoculation is indicated by a white arrow. ROI analysis shows higher signals (in % ID/cc) for MRSA 01 than seen in MRSA 03. **D**. Corresponding *ex vivo* biodistribution analysis. For MRSA 01 there was significantly higher uptake of [2-^18^F]­maltose in infected versus inflamed tissues/normal muscle. In contrast for MRSA 03 these comparisons were not significant. *P*-values were determined by two-tailed Mann–Whitney test (Graphpad Prism 10).

### The Chemoenzymatic Synthesis of [U-^13^C]­Sakebiose Represents a Promising Route to ^13^C-Enriched Disaccharides for [^13^C]­CO_2_ Breath Testing

Motivated by recent reports describing the use of ^18^F-labeled disaccharides for pathogen-specific PET imaging, we further demonstrated that reverse phosphorolysis using ^13^C-enriched sugars could be used to expand the scope of metabolites for [^13^C]­CO_2_ breath testing. Depending on the linkage type (α/β) and position, positron-labeled disaccharides could detect bacteria (α-1,3; maltose and α-1,3; sakebiose),^24^ mycobacteria (α-1,1; trehalose)^24^ or, fungi (α-1,4; cellobiose)^30^ *in vivo*. We reasoned that this synthetic strategy could afford the analogous ^13^C-enriched sugars for breath testing. Since 2-deoxy-2-[^18^F]-fluoro-sakebiose showed high consistent uptake in all *S. aureus* strains tested, including methicillin-resistant *S. aureus* (MRSA),^24^ a synthesis of [U-^13^C]­sakebiose was developed using β-d-[U-^13^C]­glucose-1-phosphate and sakebiose phosphorylase (*Anaerocolumna sp.*), identified based on its homology to other GH65 enzymes acting on α-glucosidic linkages^31^ (Figure 9A,B). Reverse phosphorolysis using β-d-[U-^13^C]­glucose-1-phosphate as the donor and d-[U-^13^C]­glucose as the acceptor was used to synthesize small quantities of universally ^13^C-enriched sakebiose to match the other probes investigated in this work. *In vitro* studies using [U-^13^C]­sakebiose showed high [^13^C]­CO_2_ production in all MRSA strains tested, in contrast to control experiments using natural abundance sakebiose or heat-killed bacteria (Figure 9C) and was stable in human serum (Supp. Figure S11). Production of ^13^C-enriched sakebiose and other “rare” disaccharides with potential pathogen specificity (i.e., trehalose, kojibiose) may be facilitated by sucrose phosphorylase variants in future work,^32^ although this strategy is best suited for the synthesis of partially enriched ^13^C-enriched metabolites.

**Figure 9:**
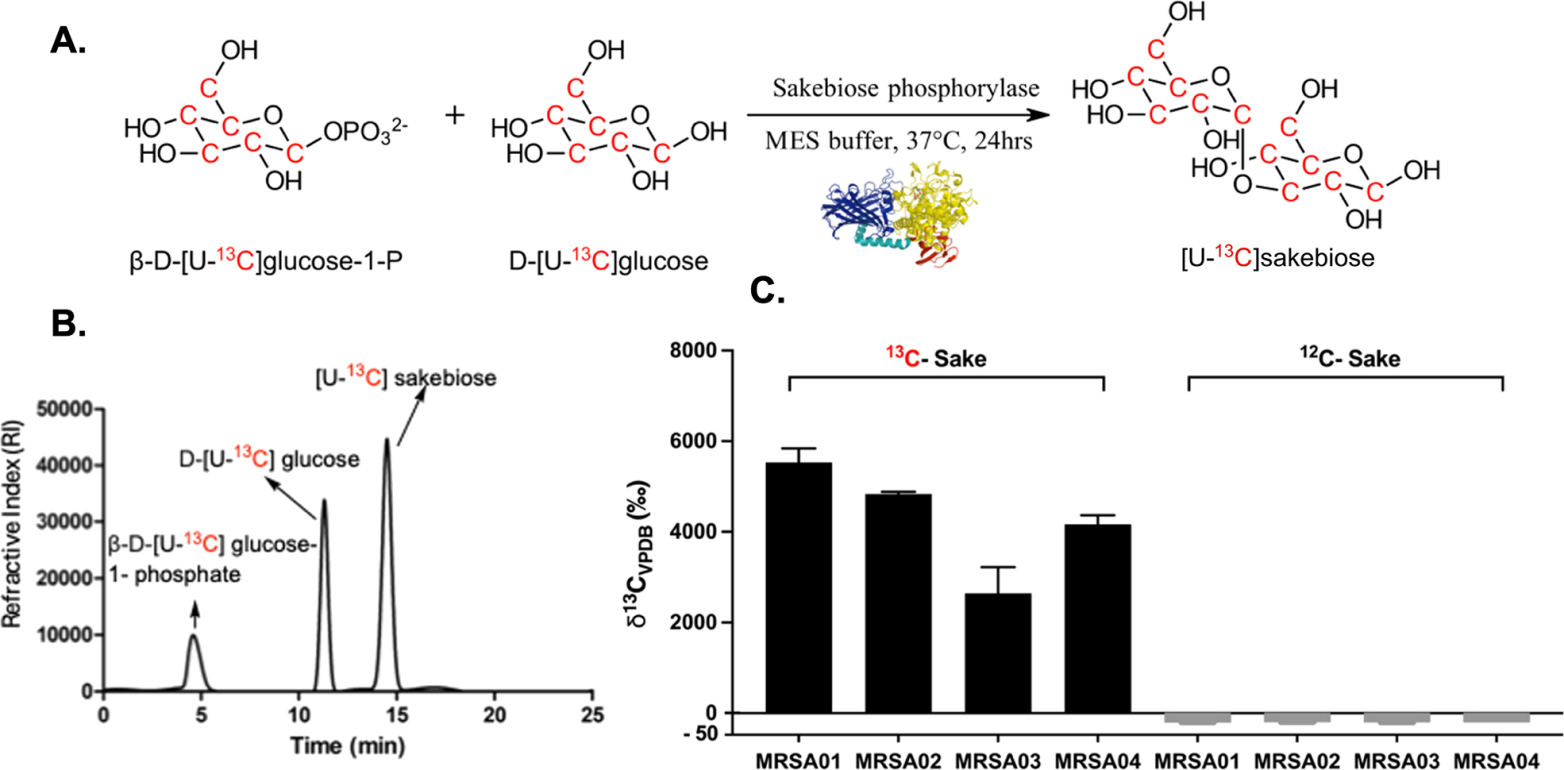
Synthesis and *in vitro* evaluation of [U**-** ^13^C]­sakebiose. **A**. Chemoenzymatic synthesis of [U-^13^C]­sakebiose (^13^C-sake) from β-d-[U-^13^C]­glucose-1-phosphate. **B**. HPLC chromatogram showing [U-^13^C]­sakebiose purification. **C**. Production of [^13^C]­CO_2_ *in vitro* was studied in methicillin-resistant *S. aureus* (MRSA). All MRSA strains studied (MRSA01-MRSA04) showed elevated production of [^13^C]­CO_2_ after incubation with ^13^C-sake, while no [^13^C]­CO_2_ production was observed when incubated with natural abundance sakebiose (^12^C-sake).

## Discussion

In this study, we explored the potential application of [^13^C]­CO_2_ breath testing to detect human bacterial infections by testing intravenously administered, bacteria-specific ^13^C-enriched metabolites. We identified several metabolites that were efficiently metabolized to [^13^C]­CO_2_ by one or more bacterial pathogens *in vitro* but produced minimal signals in uninfected animals including [U-^13^C]­maltose, d-[U-^13^C]­mannitol, and l-[U-^13^C]­arabinose. These metabolites reliably detected infection via exhaled [^13^C]­CO_2_ in several murine models of bacterial infection, including myositis, osteomyelitis, pneumonia, and bacteremia using NDIR spectroscopy. Effective response to ceftriaxone therapy of *E. coli* infections was identified using d-[U-^13^C]­mannitol highlighting a potential clinical application of this technique, to monitor treatment response and determine duration of therapy.

This diagnostic concept was motivated by recently developed PET tracers targeting bacterial metabolism including the ^18^F-radiolabeled analogs [2-^18^F]­maltose, d-[2-^18^F]­mannitol and l-[2-^18^F]­arabinose.^8,24,33,34^ The bacterial metabolism of maltose and its related α-1,4 linked oligosaccharides is mediated by the maltodextrin ABC transporter, representing an important source of glucose for bacteria^35,36^ and a way to identify infection with high specificity *in vivo* based on negligible mammalian uptake. This metabolic difference was recently exploited by Ning et al. for both optical imaging and PET via synthesis of maltodextrin-based imaging probes labeled with either a fluorescent dye^37^ or fluorine-18.^38^ Numerous other maltodextrin transporter-targeted agents were subsequently reported for imaging bacterial infection including [6-^18^F]­maltose^34^ and [6”-^18^F]­maltotriose^7^ for PET, and Cy7-1-maltotriose for photoacoustic imaging.^39^ A recent development was the efficient radiosynthesis of [2-^18^F]­maltose chemoenzymatically from widely available and most frequently used oncologic radiotracer [^18^F]­FDG in one step^24^ highlighting the recent interest in on-demand radiosyntheses of PET radiotracers for infection imaging.^30,40^ Similarly, a simple and automated [^18^F]­FDG-like radiosynthesis of the mannitol-derived radiotracer d-[2-^18^F]­mannitol was recently described for imaging bacterial infection.^8^ Mannitol-derived diagnostic tools target the phosphoenolpyruvate-dependent sugar phosphotransferase system of bacteria, that catalyzes both translocation across the cell membrane and phosphorylation.^41,42^ Finally, l-arabinose has been identified as a bacteria-specific substrate based on the activities of arabinose isomerase (EC 5.3.1.4), ribulokinase (EC 2.7.1.16), and ribulose-5-phosphate-4-epimerase (EC 5.1.3.4) for metabolism via the pentose phosphate pathway.^43–45^ Interestingly, the fluorine-18 labeled analog l-[2-^18^F]­arabinose^33^ did not recapitulate the high bacterial sensitivity observed for native l-arabinose, characterized using the β-particle emitting l-[1-^14^C]­arabinose. As reflected by this result, fluorine-18 labeling can significantly alter bacterial metabolism of PET tracers. In contrast, the ^13^C-enriched diagnostic tools reported in this manuscript are chemically and biochemically identical to their unlabeled counterparts, producing robust and predictable behavior in both bacteria and mammals.

Breath testing via [^13^C]­CO_2_ has the potential to complement both current and emerging diagnostic tools for the noninvasive detection of bacterial infection. Clinicians often rely on leukocyte count and differential, C-reactive protein (CRP), erythrocyte sedimentation rate (ESR), alpha-defensin, and leukocyte esterase for initial evaluation of possible infection, but these are not specific for invasive bacterial infection. Blood culture can be insensitive as many invasive bacterial infections do not cause bacteremia until late in the clinical course, if at all, and nonspecific due to procedural contaminants.^46^ Emerging molecular testing techniques including plasma metagenomic sequencing^47^ and multiplex PCR tools^48^ may have advantages in determining organism identity but limitations in quantification and infrastructure costs. A [^13^C]­CO_2_ breath testing technique could substantially improve the specificity of these widely available and emerging laboratory evaluations, differentiating patients with invasive bacterial infection from those with viral or noninfectious illnesses during early medical evaluation. Maltose, d-mannitol, and l-arabinose have all been administered safely to humans intravenously,^49–51^ indicating facile delivery of their ^13^C-enriched analogs to patients. Furthermore, portable and inexpensive ICOS and NDIR spectroscopy instruments could be readily incorporated into medical practice.

Invasive bacterial infections are often difficult to identify and treat in the clinic, due to the overlap of symptoms and laboratory values seen with viral or noninfectious mimics. This is particularly true for lung, solid organ musculoskeletal and other occult infections. Bacterial pneumonias are notoriously difficult to distinguish from viral infection, atelectasis (collapse), pulmonary edema and neoplasm.^52,53^ Since community-acquired pneumonias are frequently caused by Gram-positive organisms, [U-^13^C]­maltose and d-[U-^13^C]­mannitol might be applied to their detection as highlighted in this manuscript. Definitively diagnosing bacterial endocarditis is frequently difficult, relying on composite clinical data and blood cultures or molecular testing that require several days to interpret, and can be insensitive and nonspecific. Standard of care requires 6 or more weeks of intravenous antibiotics, and given the poor outcomes associated with delayed treatment,^54^ high-risk patients with suspected endocarditis are often treated even if cultures are negative. Patients commonly develop progressively obstructive lesions of non-native cardiac valves, which often cannot be reliably diagnosed as infectious or inflammatory with current imaging methods. Successful application of [^13^C]­CO_2_ breath testing could provide timely, definitive diagnosis of bacterial endocarditis when used in concert with microbiological and imaging testing modalities. Finally, vertebral discitis-osteomyelitis (VDO), periprosthetic joint infection (PJI), and diabetic foot infection also represent significant diagnostic challenges. For these soft tissue and bone infections, tissue/fluid sampling for culture is the diagnostic gold standard but can be insensitive in the setting of pretreatment with antibiotics, and difficult to obtain for deep-seated infections. Tissue/fluid sampling in these spaces frequently represents unacceptable risk and medical cost. In general, structural imaging techniques are limited in the accurate diagnosis of these orthopedic infections, as plain films, computed tomography (CT) and magnetic resonance imaging (MRI) all rely on the presence of nonspecific structural abnormalities that often occur late in the disease process and can mimic processes such as osteonecrosis, rheumatologic disease, or age-related degeneration. New, specific point-of-care methods to detect bacterial infection and distinguish from nonbacterial processes would be revolutionary in the acute care setting, potentially decreasing the time to diagnosis of invasive bacterial infection, and reducing unnecessary hospitalizations and antibiotic use. Furthermore, reliable tools to trend response to antimicrobial treatment and resolution of infection would markedly improve clinical management, patient outcomes, and medical and financial burden.

The production of bacteria-specific [^13^C]­CO_2_ signals in preclinical myositis, bacteremia, pneumonia and osteomyelitis models suggest that identifying these infections in humans is feasible. The next steps are to test promising ^13^C-enriched metabolites in uninfected control subjects and clinical infections, to establish robust, bacteria-dependent [^13^C]­CO_2_ production. These studies will help us to understand which ^13^C-enriched metabolites are best for various infections. We do not know at present whether certain ^13^C-enriched metabolites will show too much background in normal human subjects, or which will be most sensitive for Gram-positive or Gram-negative infections in man. Based on data in the current manuscript, we propose that (1) ^13^C-enriched metabolites will be used in humans depending on their organism sensitivity and (2) following known infections to resolution will be a powerful application of this technique. In the acute setting, the use of a specific ^13^C-enriched metabolite might follow the likelihood of certain causative organisms. Vertebral discitis-osteomyelitis is most frequently caused by staphylococcal species^55^ and other Gram-positive bacteria, so our data supports the use of [U-^13^C]­maltose when this infection is suspected. Similarly, infections of the kidneys and hepatobiliary system^56,57^ are frequently caused by Gram-negative species so d-[U-^13^C]­mannitol would be used.

The current study was performed using commercially available ^13^C metabolites, but reported *in vitro* studies indicate other metabolites with pathogen-specific uptake/metabolism that warrant consideration.^5^ For example, β-linked ^13^C-enriched oligosaccharides could potentially be used in fungal detection,^30^ to complement the β-1,3-d-glucan test already used to detect invasive fungal disease.^58^ Synthesis of these and other metabolites including the expensive [U-^13^C]­maltotriose may be facilitated by newer chemoenzymatic techniques,^32,59^ potentially producing a class of pathogen-specific diagnostic agents at much lower cost. A recent study used the sucrose phosphorylase variant (L341I_Q345S) to produce >3 kg of the “rare” disaccharide kojibiose (α-1,2 linked),^32^ representing a path to the syntheses of pathogen-specific, ^13^C-enriched sugars. A [^13^C]­CO_2_ breath testing approach could also be applied to a broad spectrum of pathologies, including detecting neoplasia noninvasively, as cancer-specific pathways are elucidated. There have been numerous efforts to diagnose neoplasms (especially lung) via exhalation of volatile organic compounds,^60,61^ with the recently reported ethyl-β-d-glucuronide-d_5_ reporter^62^ highlighting the use of enriched metabolites in this context. Minimal or absent background signals are a basic requirement for many diagnostic approaches. In a pilot experiment, we studied mice with prostate cancer (22Rv1) xenografts in a metabolic chamber following intravenous [1-^13^C]­glycine administration (Supp. Figure S14). Prostate cancer cells have high levels of glycine decarboxylase (EC 1.4.4.20) an enzyme shown to regulate invasion, metastases and immune escape.^63^ Tumor mice exhaled more [^13^C]­CO_2_ than their wild-type counterparts, but this elevation was subtle based on high background signals. This study highlights both the possibilities and limitations of using metabolic derangements to diagnose human diseases.

Several challenges in using the proposed [^13^C]­CO_2_ breath testing approach to diagnose bacterial infections will be addressed in future clinical trials. These pitfalls include the (largely unstudied) bacterial burden in clinically active infections, the requirement for metabolically active bacteria that may prohibit microbial detection in sessile lesions (i.e., biofilms),^64^ potential background signal from colonizing bacteria in the gut and upper respiratory tract, and limited understanding of the ^13^C-enriched metabolite doses required. From a practical standpoint, there are also significant cost and regulatory considerations. For studies in human subjects, current good manufacturing practice (cGMP) compliant materials will be required. We anticipate that the cost of cGMP-compliant ^13^C-enriched metabolites will decrease significantly once the demand for them, and supplier interest increases. In the hyperpolarized ^13^C-MRI field, the cost of various ^13^C-enriched, cGMP metabolites ([1-^13^C]­pyruvic acid, [2-^13^C]­pyruvic acid and others) has decreased significantly (>50%) over the last several years reflecting the feasibility of, and interest in human studies.^65^

Despite these concerns, optimism is warranted on several fronts. Recent data has indicated that bacteria in quiescent states still accumulate metabolism-targeted PET tracers, as recently shown for [^11^C]­PABA in an infected orthopedic implant model.^66^ Regarding dose, breath testing using [^13^C]­urea for detection of *H. pylori* has been incredibly sensitive and robust, even when low doses (20–100 mg) are used.^67,68^ Furthermore, there are potential pharmacokinetic advantages in humans, most notably in the use of d-[U-^13^C]­mannitol and similar metabolites. The biologic half-life of d-mannitol in patients with normal renal function is about 0.5–2.5 h, and it is largely unchanged when excreted in the urine- motivating its current clinical use with very high doses as an osmotic agent in treating patients with increased intracranial pressure. Therefore, d-[U-^13^C]­mannitol would be anticipated to have adequate exposure to *in vivo* pathogens and be biologically inert (no exhaled [^13^C]­CO_2_) in uninfected patients. Potential clinical use of d-[U-^13^C]­mannitol also highlights the low toxicity of the proposed ^13^C-enriched metabolites. A 2 mg dose of d-[U-^13^C]­mannitol administered to a 20-g mouse represents 100 mg/kg. Based on a frequently used allometric scaling method (that uses surface area),^69^ the corresponding human dose would be 100/12.3 or ∼ 8 mg/kg. The typical dose of intravenous mannitol for elevated intracranial pressure is 250 mg/kg to 1000 mg/kg.^70^ Similarly, maltose has been administered intravenously up to 250 mg/kg.^71^ Finally, detection of exhaled [^13^C]­CO_2_ will be improved for humans versus mice. Humans can intentionally exhale generated [^13^C]­CO_2_, producing more signal than mice passively breathing into a metabolic chamber. These considerations will be addressed in the future application of [U-^13^C]­maltose, d-[U-^13^C]­mannitol, and l-[U-^13^C]­arabinose to humans, targeting a variety of clinically challenging infections.

In conclusion, we exploited recent advances in pathogen-targeted diagnostics to detect infection noninvasively using [^13^C]­CO_2_ breath testing. The results presented represent a first step to expand the arsenal of ^13^C-enriched metabolites with potential bacterial detection specificity, better understand their bacterial conversion, and identify sensors without background signals in uninfected patients. We anticipate that this approach could also be useful in the detection of cancer and other diseases, as a safe and potentially straightforward way to identify metabolic abnormalities.

## Materials and Methods

### Study Design

This study included both *in vitro* and *in vivo* experiments to investigate the feasibility of [^13^C]­CO_2_ breath testing for detecting bacterial infections. *In vitro* assays included the screening of eight ^13^C-enriched metabolites against a panel of six clinically relevant human bacterial pathogens. To identify those metabolites selectively metabolized by bacteria, [^13^C]­CO_2_ production was detected via NDIR. Promising metabolites were then tested in murine models of infection, including myositis, pneumonia, bacteremia and osteomyelitis- by administering the candidate ^13^C-enriched compounds intravenously and measuring exhaled [^13^C]­CO_2_. Uninfected mice and mice administered natural abundance compounds served as controls to assess specificity. The study also investigated the sensitivity of this modality and its potential use in following antibiotic response in an *E. coli*-infected cohort treated with ceftriaxone. Lastly, [^13^C]­CO_2_ production by several *S. aureus* clinical strains using [U-^13^C]­maltose were correlated with retention of the analogous PET tracer [2-^18^F]­maltose.

### *In vitro* studies

#### [U-^13^C] Metabolites

Except for [U-^13^C]­sakebiose, all universally ^13^C-enriched compounds were obtained from Omicron Biochemicals (South Bend, IN) or Sigma-Aldrich (Burlington, MA) and used without further purification. [U-^13^C]­sakebiose was synthesized via reverse phosphorolysis from β–d-[U-^13^C]­glucose and β-d-[U-^13^C]­glucose-1-phosphate, which was produced via a reported method.^24^ The enzyme used was the sakebiose phosphorylase A1NP (*Anaerocolumna sp.,* Genbank ID: WOO36257.1) After completion of the reaction, [U-^13^C]­sakebiose was isolated via semipreparative HPLC purification (polyamine II; isocratic 73:27 MeCN/H_2_O; 3–4 mL min^–1^; RI detection). Product-containing fractions were pooled, concentrated to remove MeCN, and lyophilized to afford [U-^13^C] sakebiose, as confirmed by HPLC analysis, ^1^H/^13^C NMR and high-resolution mass spectrometry (HRMS).

#### [^13^C]­CO_2_ Production

*Staphylococcus aureus*, S. aureus Xen36, MRSA, *Escherichia coli*, *Salmonella typhimurium*, *Staphylococcus epidermidis*, *Enterococcus faecalis* and *Enterobacter cloacae* were grown overnight in LB in a shaking incubator at 37 °C. Overnight cultures (1.6 mL) were diluted and grown to exponential phase inside Exetainer 12 mL vials 439W/NP (Labco, Lampeter, UK). Bacterial cultures were then incubated with increasing concentrations (25, 50, 100, 1000 μM) of different ^13^C-enriched metabolites (Table S3) at 37 °C for 100 min. MRSA cultures were treated in identical fashion and incubated with 1000 mM [U-^13^C]­sakebiose After incubation, samples were frozen at −80 °C followed by determinations of δ^13^CO_2_ in the vial headspace. All samples were anonymized (no identifying information) prior to analysis, and each vial was measured using a NDIR ^13^C analyzer (Helifan FanCi; Campro Scientific) following published protocols.^72,73^ Internal ^13^CO_2_ calibrations were conducted before each round of measurements and vials containing externally validated laboratory standard gases were run in triplicate in series for every *n* = 12 breath samples. The δ^13^C values are reported in terms of ^13^C_VPDB_ ^74,75^ and all measurements were made within 4 weeks of collection.^76^

#### [2-^18^F]­Maltose Uptake

These studies were performed as previously described.^24^ Four different strains of methicillin-resistant *Staphylococcus aureus* (MRSA) were grown overnight in lysogeny broth (LB) in a shaking incubator at 37 °C (New Brunswick, Innova 42, Germany). Overnight cultures were diluted to an optical density at 600 nm (OD_600_) of 0.05 and grown to exponential phase (∼0.4–0.6). For uptake studies, bacterial cultures were then incubated with 24 μCi of [2-^18^F]­maltose at 37 °C for 90 min. After tracer incubation, bacterial cultures were centrifuged (13,200 rpm, 6 min) and washed with phosphate buffered saline (PBS). Retained radiotracer in both pellet and supernatant, was counted using an automated gamma counter (Hidex, Turku, Finland).

### *In Vivo* Studies

#### General

All animal procedures were approved by Institutional Animal Care and Use Committee (UCSF, authorization protocol number AN205622) or Animal Care and Use Committee (St. Jude Children’s Research Hospital, (SJCRH, authorization protocol number 3176)) and performed in accordance with UCSF/SJCRH guidelines regarding animal housing, pain management, and euthanasia. Unless stated otherwise, all animals have access to food and water *ad libitum*.

#### Myositis Model

Healthy mice, CBA/J (*N* = 32), C57BL/6J (*N* = 4) and NSG (*N* = 4), were used to study the baseline metabolism of the different ^13^C-enriched metabolites used in the study. For studies with [U-^13^C]­maltose and [U-^13^C]­maltotriose, the α-glucosidase inhibitor voglibose was administered simultaneously as previously described^24^ due to the presence of α-glucosidases in mice but not human sera (Supp. Figure S7–S10). The following doses were used: [U-^13^C]­maltose (2 mg, 1 mg voglibose/animal), d-[U-^13^C]­mannitol (2 mg/animal), l-[U-^13^C]­arabinose (2 mg/animal), d-[U-^13^C]­sorbitol (2 mg/animal), d-[U-^13^C]­glucose (2 mg/animal), [^13^C]­urea (2 mg/animal), [U-^13^C]­maltotriose (2 mg, 1 mg voglibose/animal), d-[U-^13^C]­xylose (2 mg/animal) and [1-^13^C]­glycine (3 mg/animal). All doses were prepared in saline (100 μL/animal). After metabolite administration via intravenous (IV) injection the mice were quickly relocated to individual 1-L metabolic chambers attached to a positive/negative pump (Fristaden Lab) for the duration of the experiment.^77^ The metabolic chambers were periodically sealed for 2 min to allow the CO_2_ levels to increase at 0-, 10-, 20-, 40-, 80-, and 100 min postmetabolite injection. A 6 mL subsample of the gas inside each metabolic chamber was then transferred by negative pressure into a previously evacuated 12 mL vial. Baseline breath samples were also collected before ^13^C-metabolite administration. The breath samples were frozen at −80 °C until [^13^C]­CO_2_ analysis as described above.

To evaluate the production of [^13^C]­CO_2_ by the bacteria during an active infection, *S. aureus* and *E. coli* were used to induce a hind limb myositis in 24 CBA/J mice.^6^ Both strains were grown overnight at 37 °C and diluted in saline to obtain the desired bacterial load for infection (∼7 × 10^7^ CFU). At 12 h post infection [U-^13^C]­maltose (2 mg, 1 mg voglibose/animal), d-[U-^13^C]­mannitol (2 mg/animal) or l-[U-^13^C]­arabinose (2 mg/animal) were intravenously administered and studied following the above-mentioned protocol. Alternatively, natural abundance maltose (2 mg, 1 mg voglibose/animal), d-mannitol (2 mg/animal) or l-arabinose (2 mg/animal) was intravenously administered as a control.

For fasting studies, 8 CBA/J mice (*N* = 4 healthy, *N* = 4 *E. coli* myositis) were fasted for 12 h prior to IV administration of d-[U-^13^C]­mannitol (2 mg/animal; 100 μL).

For treatment studies, Ceftriaxone (Sigma) was administered subcutaneously every 90 min (5 mg/kg) for 24 h.

After breath testing studies, infected tissue of euthanized mice was excised and embedded in optimum cutting temperature (OCT) medium at −80 °C overnight. Afterward, 10 μm thick sections were cut and stained following Gram staining protocol (American Society for Microbiology (ASM)). Muscle sections were imaged with a Nikon Eclipse Ti microscope (Nikon Instruments Inc.). Infected tissue was otherwise harvested and homogenized to determine colony forming units (CFU). Homogenized infected muscle was serially diluted and plated onto LB agar plates and incubated at 37 °C overnight followed by counting.

#### Bacteremia Model

*E. coli* was used to induce bacteremia in 16 CBA/J mice. *E. coli* was grown in LB overnight at 37 °C and diluted in saline to obtain the desired bacterial load for infection (∼10^6^ CFU) and injected via intraperitoneal (IP) injection. At 12 h post infection d-[U-^13^C]­mannitol (2 mg/animal) or l-[U-^13^C]­arabinose (2 mg/animal) were intravenously administered and studied following the above-mentioned protocol. Alternatively, natural abundance d-mannitol or l-arabinose were intravenously administered as a control. All ^13^C-metabolite doses were prepared in saline (100 μL/animal).

To determine CFU, liver, kidneys and spleen were excised, homogenized, serially diluted, plated onto LB agar plates and counted.

#### Pneumonia Model

Healthy C57BL/6J mice (*N* = 4) were used to study the baseline metabolism of [U-^13^C]­maltose (2 mg, 1 mg voglibose/animal) following the above-mentioned protocol. To induce lung infection, *S. aureus* Xen36 (ATCC 49525 with *Photorhabdus luminescens luxABCDE* operon, PerkinElmer, USA) was grown overnight in Todd-Hewitt broth supplemented with 0.2% yeast extract. Bacteria were grown overnight at 37 °C and diluted in saline to obtain the desire bacteria load for inoculation (∼10^7^ CFU). C57BL/6J mice (*N* = 12) were anesthetized with 3% isoflurane (Dechra Pharmaceuticals, England) and inoculated with 100 μL (∼10^7^ CFU) intranasally.^29^ 24 h post infection, [U-^13^C]­maltose (2 mg, 1 mg voglibose/animal, 100 μL saline) was intravenously administered and studied following the same breath testing protocol. Heat-killed control samples were prepared by incubating the bacterial cultures at 90 °C for 30 min prior intranasal administration. To determine CFU, lungs were excised, homogenized, and serially diluted. The diluted samples were plated onto LB agar plates, and the number of colonies was then counted and used to calculate the CFU per unit volume.

#### Osteomyelitis Model

*S. aureus* Xen36 was grown following the same protocol as for lung inoculations. C57BL/6J mice (N = 16) were given 2 mg/mL of Metacam (Wedgewood Connect) and 1.3 mg/mL of Ethiqa (Fidelis) subcutaneously 20 min prior to infection. Mice were anesthetized with 3% isoflurane. Injections were done by plucking the hair from the patella, cleansing the area with aseptic technique, and bone defects created at the distal femur by trephination with a 25-gauge needle. Mice were then inoculated intrafemorally with 2 μL of bacteria (∼4x 10^7^ CFU) delivered through the bone defect into the intramedullary canal. Post infection, mice were given Metacam every 24 h for 72 h post infection and supplemental care. Mice femurs were harvested at 7dpi for microCT scans. At days 4 and 8 post infection, [U-^13^C]­maltose was intravenously administered and studied following the same breath testing and CFU protocol. μPET/CT using [2-^18^F]­maltose, MRI, and high-resolution CT of this osteomyelitis model were obtained as follows.

#### PET/CT (Osteomyelitis)

At day 7 postinfection, mice were anesthetized in a chamber (3% isoflurane in air delivered at 0.5 L/min) and maintained using nose-cone delivery (1–2% isoflurane in air delivered at 0.5 L/min). Catheters filled with saline were placed in the tail veins of 3 mice, which were all placed on a 3-mouse multimodal bed. The mice were then transferred to an Si78 μPET/CT scanner (Bruker Biospin GmbH, Ettlingen, Germany) and were provided thermal support using air heated using warm water as a heat exchange and a physiological monitoring system to monitor breath rate. The mice were then studied using whole-body CT, which was taken for anatomical correlation and attenuation correction. Next, a 120 min dynamic PET scan was done with the mice injected about 15 s after the start of the scan. Radiosynthesis of [2-^18^F]­maltose from 2-deoxy-2-[^18^F]­fluoro-d-glucose was performed as reported previously via reverse phosphorolysis using maltose phosphorylase (Sigma-Aldrich).^24^ The mice were injected with [2-^18^F]­maltose (∼100 μL, 138–177 μCi, 1 mg of voglibose) followed by 60 μL of saline to push through remaining activity in the catheter. The PET was reconstructed with a 0.5 mm Maximum Likelihood Expectation Maximum (MLEM) algorithm. The resulting PET-CT images were analyzed with Pmod (Pmod Technologies LLC).

#### MRI (Osteomyelitis)

Mice (*N* = 3) were also studied via magnetic resonance imaging (MRI) on day 7 postinfection. MRI was performed on a Bruker Biospec 94/30 MRI system (Bruker Biospin MRI GmbH, Ettlingen, Germany). Prior to scanning, mice were anesthetized in a chamber (3% Isoflurane in air delivered at 0.5 L/min) and maintained using nose-cone delivery (1–2% Isoflurane in air delivered at 0.5 L/min). MRI was acquired with an 86 mm transmit/receive coil. After the localizer, a T_2_-weighted Rapid Acquisition with Refocused Echoes (RARE) sequence was performed on each mouse separately in the axial orientation (TR/TE = 2000/8.5 ms, matrix size = 128 × 128, field of view = 25 mm × 25 mm, slice thickness = 1.0 mm, number of slices = 23, number of averages = 8) with a saturation pulse over the other two mice to prevent wrap-around artifacts.

##### High-Resolution CT (Osteomyelitis)

High-resolution CT was performed on a Bruker Skyscan 1276 system (Bruker Biospin GmbH, Ettlingen, Germany) on day 8 postinfection following animal sacrifice. The infected femur (rear left) was wrapped in gauze and taped to a 12 mm diameter cassette. The cassette was placed in the scanner, and a scout scan was taken to locate the femur. The CT scan was taken over 2 bed positions and had the following parameters: 70 kV voltage, 200 μA current, 0.5 mm Al filter, 0.2° rotation steps over 360°, and 2 frame averaging. The total acquisition time was about 2 h, and the reconstructed image had 3 μm isotropic resolution.

#### Tumor Xenograft Model

NOD SCID gamma (NSG) mice (N = 4) were used to study the baseline metabolism of [1-^13^C]­glycine (3 mg/animal) following the mentioned breath testing protocol. Mice ages 6–8 weeks old were then inoculated intravenously with 1 million 22Rv1-Luc cells in PBS (100 mL).^78^ Bioluminescence imaging (BLI) was used to monitor graft progression. After 4 weeks, mice were administered [1-^13^C]­glycine (3 mg/animal) and studied following the same breath testing protocol.

Liver tissue of euthanized mice was excised and embedded in optimum cutting temperature (OCT) at −80 °C overnight. Afterward, 10 μm thick sections were cut and stained with hematoxylin and eosin staining protocol. Tumor sections were imaged with a Nikon Eclipse Ti microscope (Nikon Instruments Inc.).

**PET/CT (myositis)**: CBA/J mice (*N* = 8) were inoculated with MRSA (∼7 × 10^7^ CFU) in the left deltoid muscle and a 10-fold-higher bacterial load of heat-killed bacteria in the right deltoid muscle. MRSA was grown overnight at 37 °C and diluted in saline to obtain the desired bacterial load for infection. At 12 h post infection, [2-^18^F]­maltose was injected via the tail vein (∼100 μL, 200 μCi, 1 mg of voglibose). The mice were then imaged by μPET-CT (90 min dynamic PET scan, 5 min CT). The resulting μPET-CT images were analyzed with Amide’s Medical Image Data Examiner, as described below.

### Statistical Analysis

Data from *in vivo* [^13^C]­CO_2_ production studies are presented as mean and SEM and analyzed using unpaired two-tailed Student’s *t* test using GraphPad Prism 10 (GraphPad Software Inc.). *Ex vivo* biodistribution studies are represented as box plot and *P*-values were determined by two-tailed Mann–Whitney test (GraphPad Software Inc.). *P*-values < 0.05 were considered statistically significant. AMIDE open-source software^79^ or Pmod was used to analyze all PET imaging data. Volumes of interest (VOI’s) were manually drawn for each indicated organ and expressed as the percent injected dose per gram.

## Supplementary Material





## Data Availability

All data are available in the main text or in the . Reprint request to the corresponding authors.

## Notes

Detailed information regarding synthesis, *in vitro* and *in vivo* experiments not reported in the main text as well as supplementary figures (Figures S1–S12) and Tables (Tables S1–S3) (PDF)
Transparent Peer Review report available (PDF)
