# Correction to “Detecting Bacteria in Their Mammalian Hosts Using Metabolism-Targeted [^13^C]CO_2_ Breath Testing”

**DOI:** 10.1021/acscentsci.6c01043

**Published:** 2026-08-01

**Authors:** Marina López-Álvarez, Sang Hee Lee, Anju Wadhwa, Mohammad Yaqoob Bhat, Tyler S. Simmons, Jung Min Kim, Anil Bidkar, Spenser R. Simpson, Shari Dhaene, Jeffrey D. Steinberg, Joseph Blecha, Wenxing Zou, Robert R. Flavell, Marshall D. McCue, Amanda M. Green, Renuka Sriram, Tom Desmet, Joanne Engel, Jason W. Rosch, Michael A. Ohliger, Kiel D. Neumann, David M. Wilson

Wenxing Zou is being added to the authorship list. She is affiliated with Department of Radiology and Biomedical Imaging, University of California, San Francisco, San Francisco, CA 94158, United States.

