# Supplementary material for: Detecting Bacteria in Their Mammalian Hosts Using Metabolism-Targeted [13C]CO2 Breath Testing
Source: ACS Cent Sci. 2026 Mar 18;12(4):457–72. doi: 10.1021/acscentsci.5c01995 (PMC13107215; doi:10.1021/acscentsci.5c01995)
Supplement: Supplementary file 1 [file oc5c01995_si_001.pdf]

Supplementary Information

Title: Detecting bacteria in their mammalian hosts using metabolism-targeted [^{13}C]CO_2 breath testing

Marina López-Álvarez[1], Sang Hee Lee[1], Anju Wadhwa[1], Mohammad Yaqoob Bhat[1], Tyler S. Simmons[2], Jung Min Kim[1], Anil Bidkar[1], Spenser R. Simpson[3], Shari Dhaene[4], Jeffrey D. Steinberg[5], Joseph Blecha[1], Robert R. Flavell[1], Marshall D. McCue[6], Amanda M. Green[7], Renuka Sriram[1] Tom Desmet[4], Joanne Engel[8], Jason W. Rosch[2], Michael A. Ohliger[1,9], Kiel D. Neumann[3,10*], and David M. Wilson[1*]

[*]Correspondence and Reprint Request:

Kiel D. Neumann, Ph.D.
Department of Diagnostic Imaging
St. Jude Children's Research Hospital
Memphis, TN 38105
Phone: (901) 595-2119
kiel.neumann@stjude.org

David Wilson, M.D., Ph.D.
Department of Radiology and Biomedical Imaging
University of California, San Francisco
San Francisco, CA 94143
Phone: (415) 353-1668
david.m.wilson@ucsf.edu

First Author:

Marina López-Álvarez
Department of Radiology and Biomedical Imaging
University of California, San Francisco
180 Berry St.
San Francisco, CA 94107
marina.lopezalvarez@ucsf.edu

Table of Contents

A.	Supplemental Figures…………………………………………………………………	2
B.	Synthetic Procedures…………………………………………………………………	13
C.	In vivo Procedures……………………………………………………………………	16
D.	References ………………………………………………………………………………	10

A. Supplemental Figures:

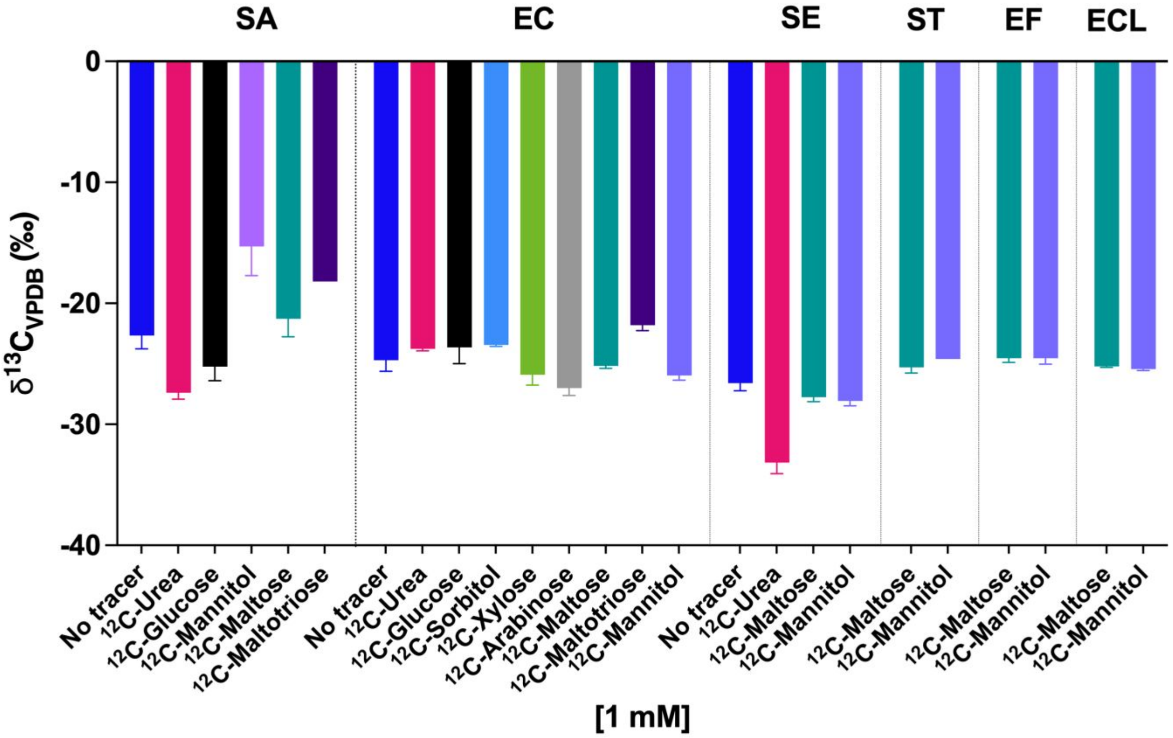

Figure S1. *In vitro* [^{13}C]CO_2 production by *S. aureus* (SA), *E. coli* (EC), *S. typhimurium* (ST), *E. faecalis* (EF) and *E. cloacae* (ECL) after incubation with natural abundance compounds. All cultures demonstrated no production of [^{13}C]CO_2 (See **Figure 2** for comparison).

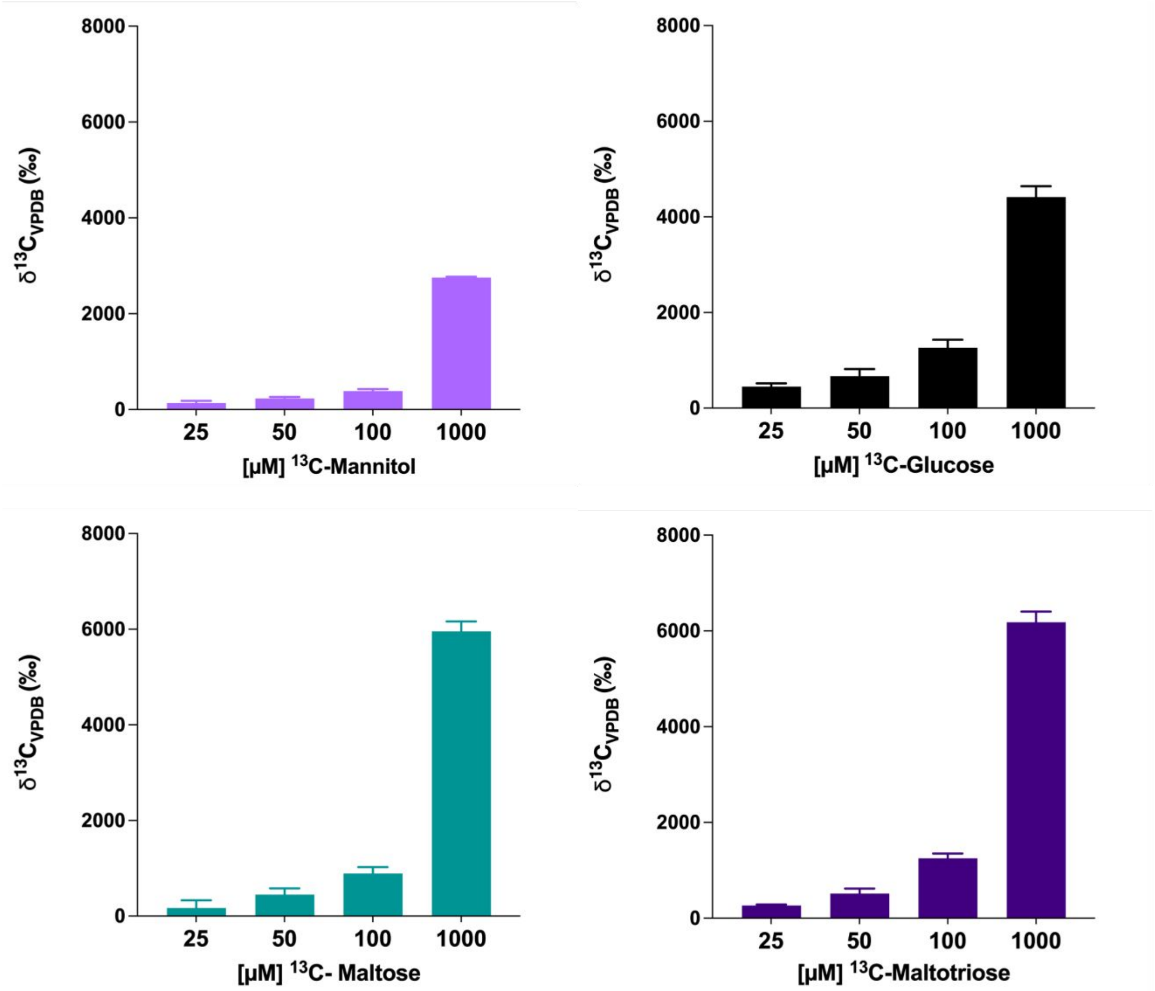

Figure S2. *In vitro* [^{13}C]CO_2 production by *S. aureus* after incubation with increasing concentrations of [U-^{13}C]maltose, [U-^{13}C]maltotriose, [U-^{13}C]mannitol, and D-[^{13}C]glucose. As expected, higher concentrations of sugar led to higher [^{13}C]CO_2 production.

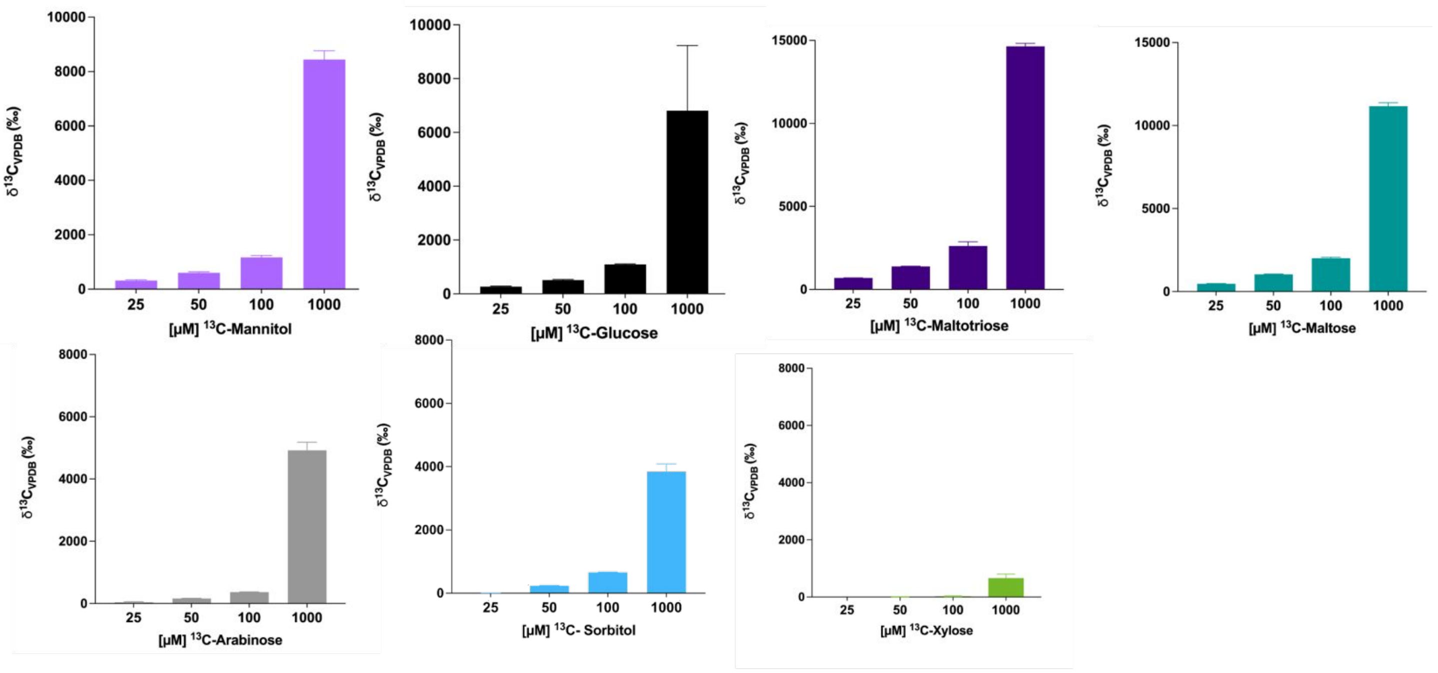

Figure S3. *In vitro* [^{13}C]CO_2 production by *E. coli* after incubation with increasing concentrations of [U-^{13}C]maltose, [U-^{13}C]maltotriose, [U-^{13}C]mannitol, D-[^{13}C]glucose, L-[U-^{13}C]arabinose, D-[U-^{13}C]sorbitol and D-[U-^{13}C]xylose. All compounds with the exception of D-[U-^{13}C]xylose showed high production of [^{13}C]CO_2 by *E. coli* after incubation at a concentration of 1 mM.

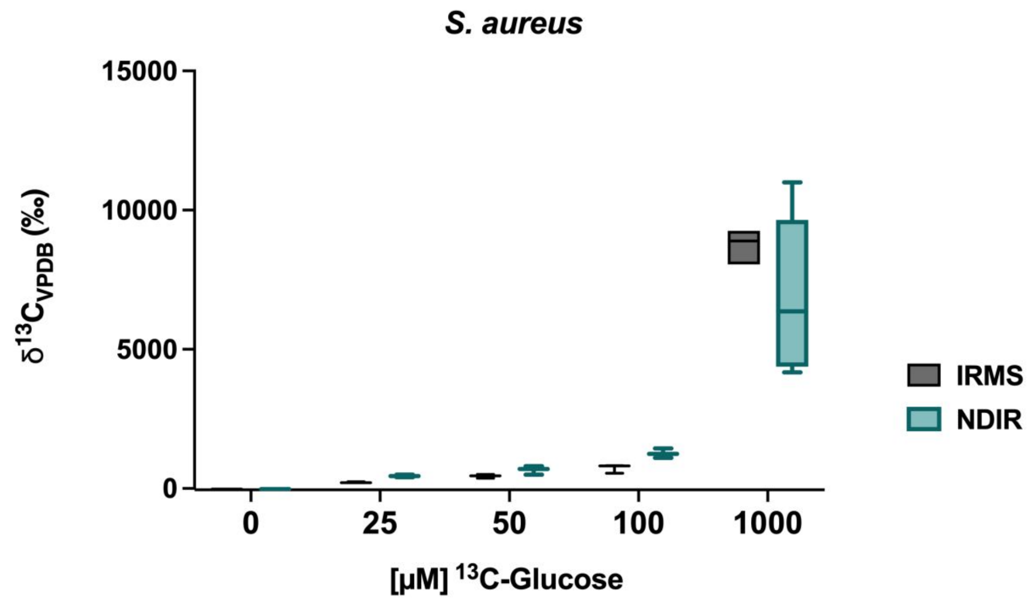

Figure S4. **Comparison between IRMS and NDIR spectroscopy [^{13}C]CO_2 detection**. *S. aureus* cultures were treated with D-[^{13}C]glucose as above, and samples were sent for both IRMS (UC Davis Mass Spectroscopy facility) and NDIR [^{13}C]CO_2 detection. The two techniques yielded comparable $\Delta\ \delta^{13}C_{VPDB}$ (‰).

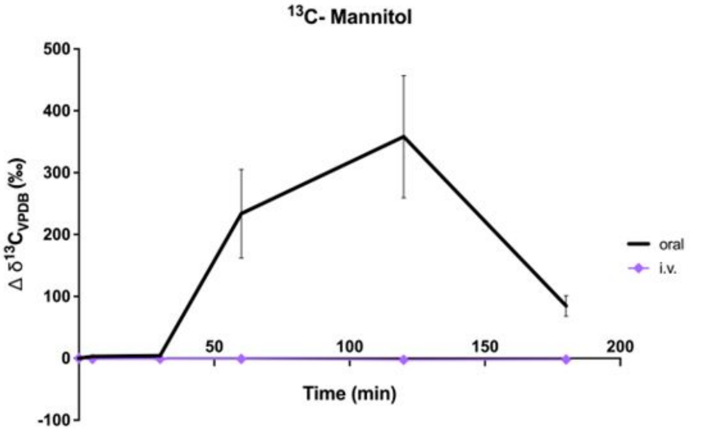

Figure S5. *In vivo* [^{13}C]CO_2 production in healthy mice after oral administration of D-[U-^{13}C]mannitol (N=4). As expected, high [^{13}C]CO_2 production was observed after oral administration of ^{13}C]mannitol due to the presence of bacteria (i.e. *E. coli*) in the gastrointestinal track.

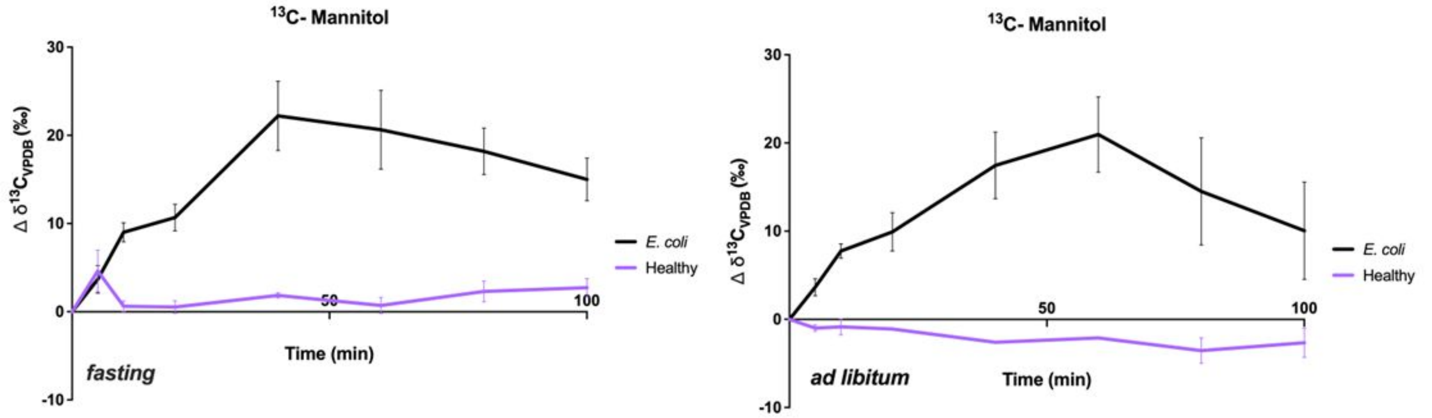

Figure S6. Comparison between *in vivo* [^{13}C]CO_2 production in mice under fasting or *ad libitum* conditions. Similar [^{13}C]CO_2 production was observed CBAJ mice with unlimited access to food and water (ad libitum) or mice fasted for 12 hours. (N=4 per group)

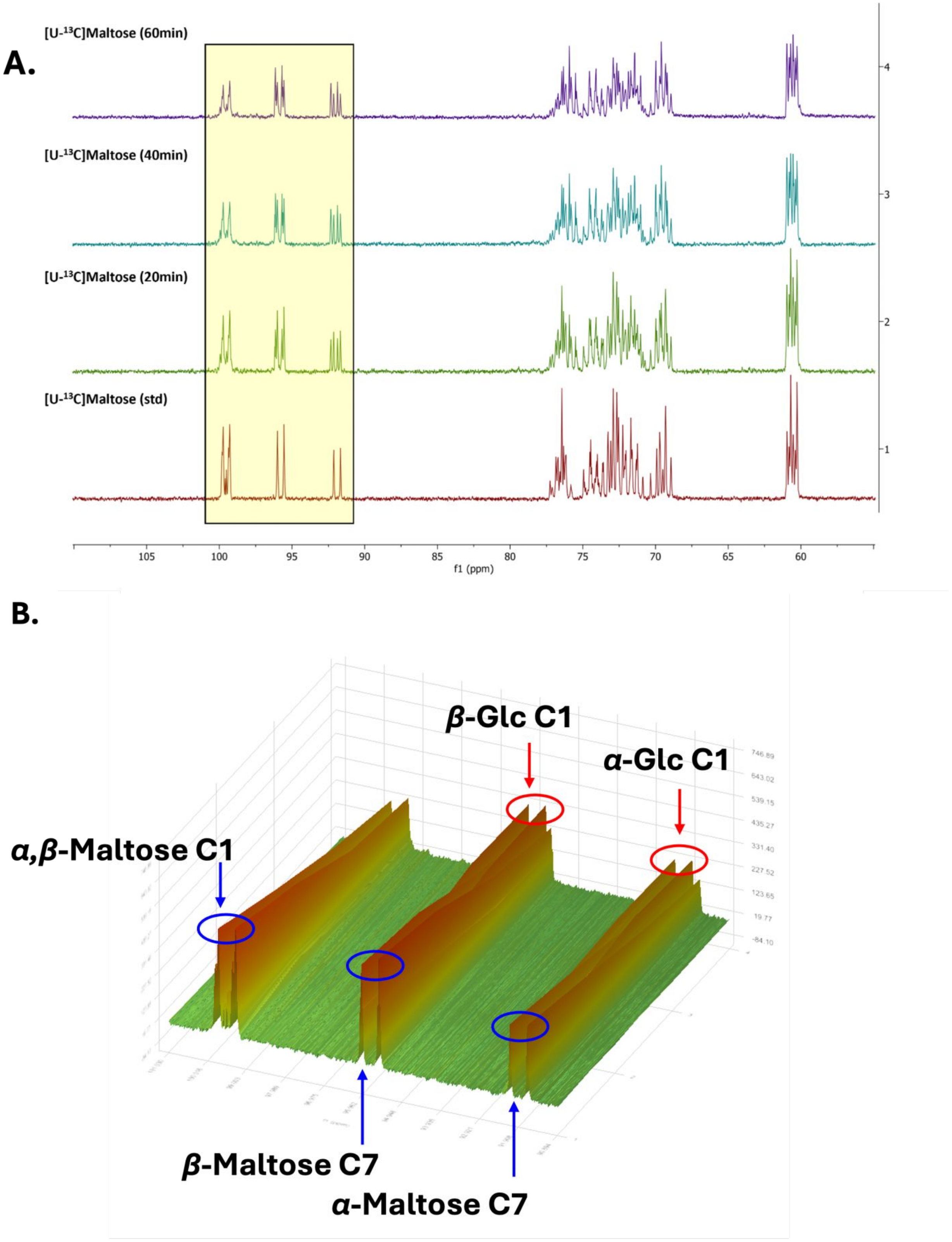

Figure S7. ***In vitro* serum stability of [U-^{13}C]maltose in mouse serum without α-glucosidase inhibitor. A.** The 100 MHz ^{13}C NMR spectrum of [U-^{13}C]maltose in D_2O (std) and mouse serum incubated for 20, 40, and 60 min respectively. **B.** 3D-stacked ^{13}C NMR spectrum magnified between 101.0-92.0ppm. Upon incubation in mouse serum, the peak intensity of [U-^{13}C]maltose (δ 99.51 (d, *J* = 45.0 Hz, C1 of α,β-Maltose), 99.36 (d, *J* = 45.2 Hz), 95.78 (d, *J* = 45.7 Hz, C7 of β-Maltose), 91.90 (d, *J* = 45.9 Hz, C7 of α-Maltose)) were decreased

over time, while the peak intensities of D-[U-^{13}C]glucose (δ 95.87 (dt, *J* = 45.5, 4.5 Hz, C1 of β-Glc), 92.00 (dt, *J* = 45.5, 2.3 Hz, C1 of α-Glc))were increased likely due to the activity of alpha glucosidase in mouse serum.

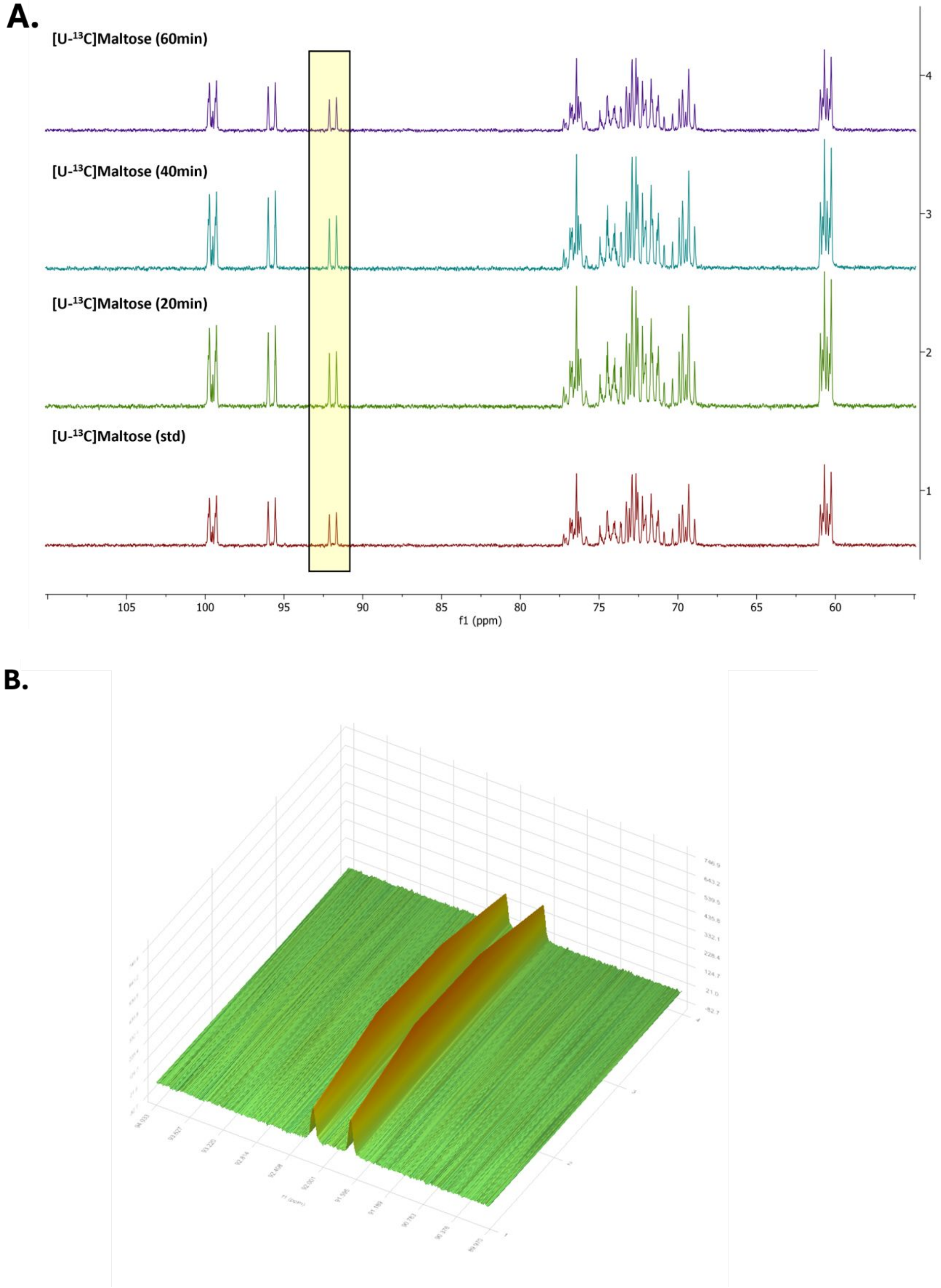

Figure S8. ***In vitro*** **serum stability of [U-^{13}C]maltose in mouse serum in the presence of α-glucosidase inhibitor voglibose. A.** The 100 MHz ^{13}C NMR spectrum of [U-^{13}C]maltose in D_2O (std) and mouse serum

incubated for 20, 40, and 60 min respectively. **B.** 3D-stacked ^{13}C NMR spectrum magnified between 98.0-91.0 ppm. The peak intensity of [U-^{13}C]maltose was stable over time in mouse serum in the presence of inhibitor.

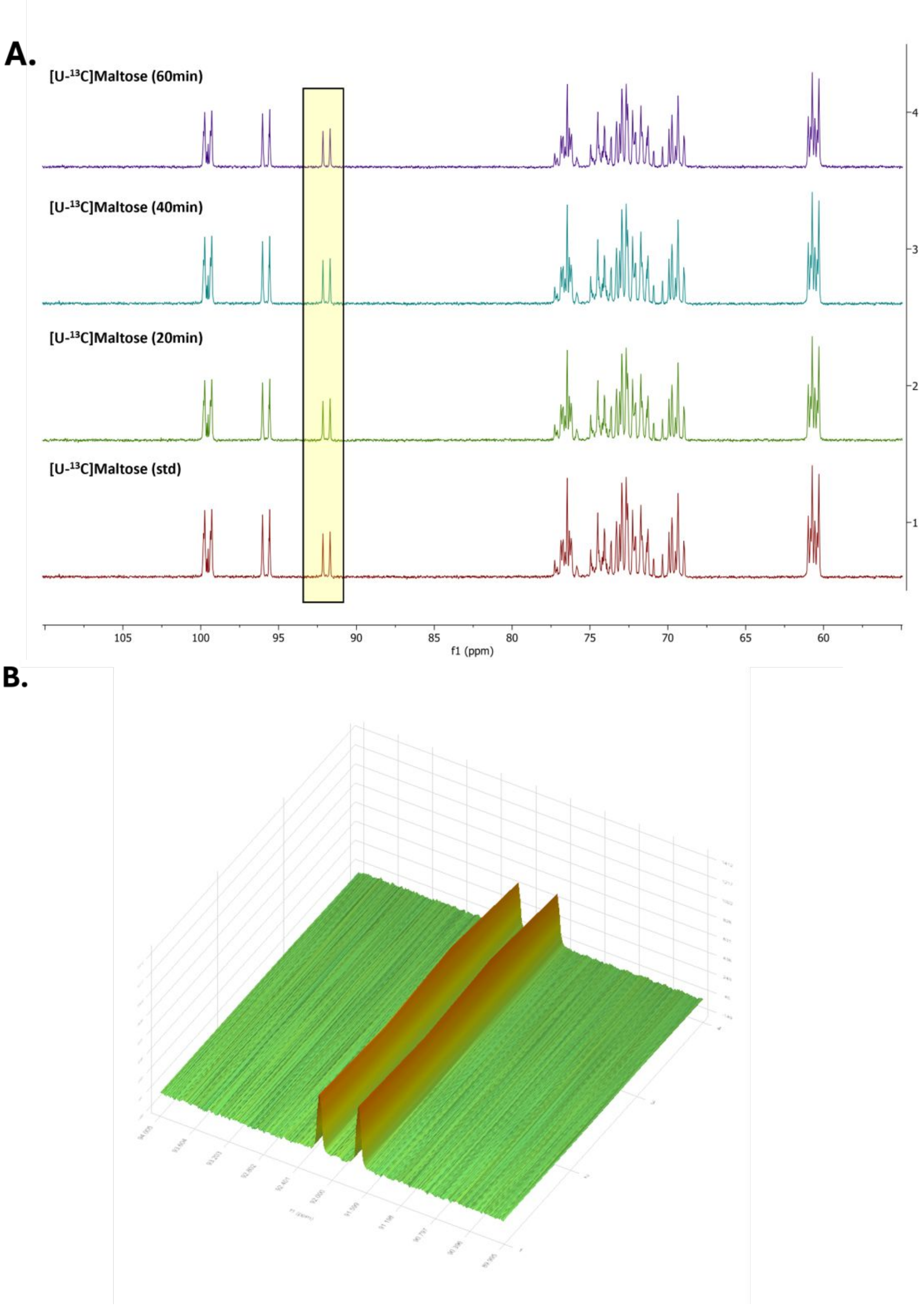

Figure S9. ***In vitro* serum stability of [U-^{13}C]maltose in human serum without α-glucosidase inhibitor. A.** The 100 MHz ^{13}C NMR spectrum of [U-^{13}C]maltose in D_2O (std) and human serum incubated for 20, 40, and 60 min respectively. **B.** 3D-stacked ^{13}C NMR spectrum magnified between 98.0-91.0ppm. The peak intensity of [U-^{13}C]maltose was intact over time due to the absence of alpha glucosidase activity.

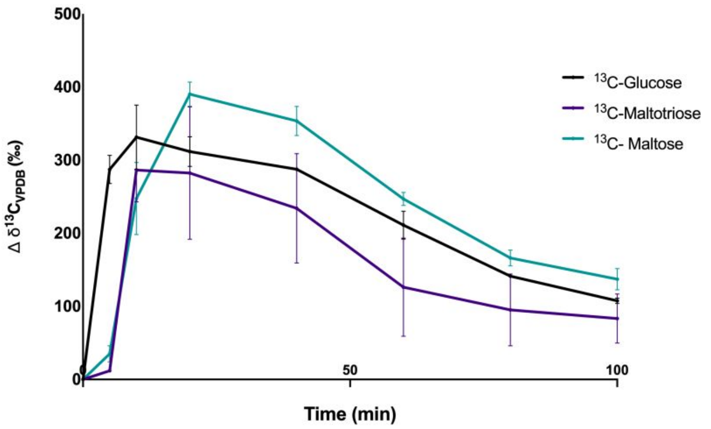

Figure S10. *In vivo* [^{13}C]CO_2 production in healthy mice after incubation with control compound D-[^{13}C]glucose and [U-^{13}C]maltotriose or [U-^{13}C]maltose without the α-glucosidase inhibitor voglibose (N=4, per group). Due to the presence of α-glucosidase enzyme in mice serum, [U-^{13}C]maltotriose and [U-^{13}C]maltose are degraded from the nonreducing end, hydrolyzing [U-^{13}C]maltotriose and [U-^{13}C]maltose to D-[^{13}C]glucose[1].

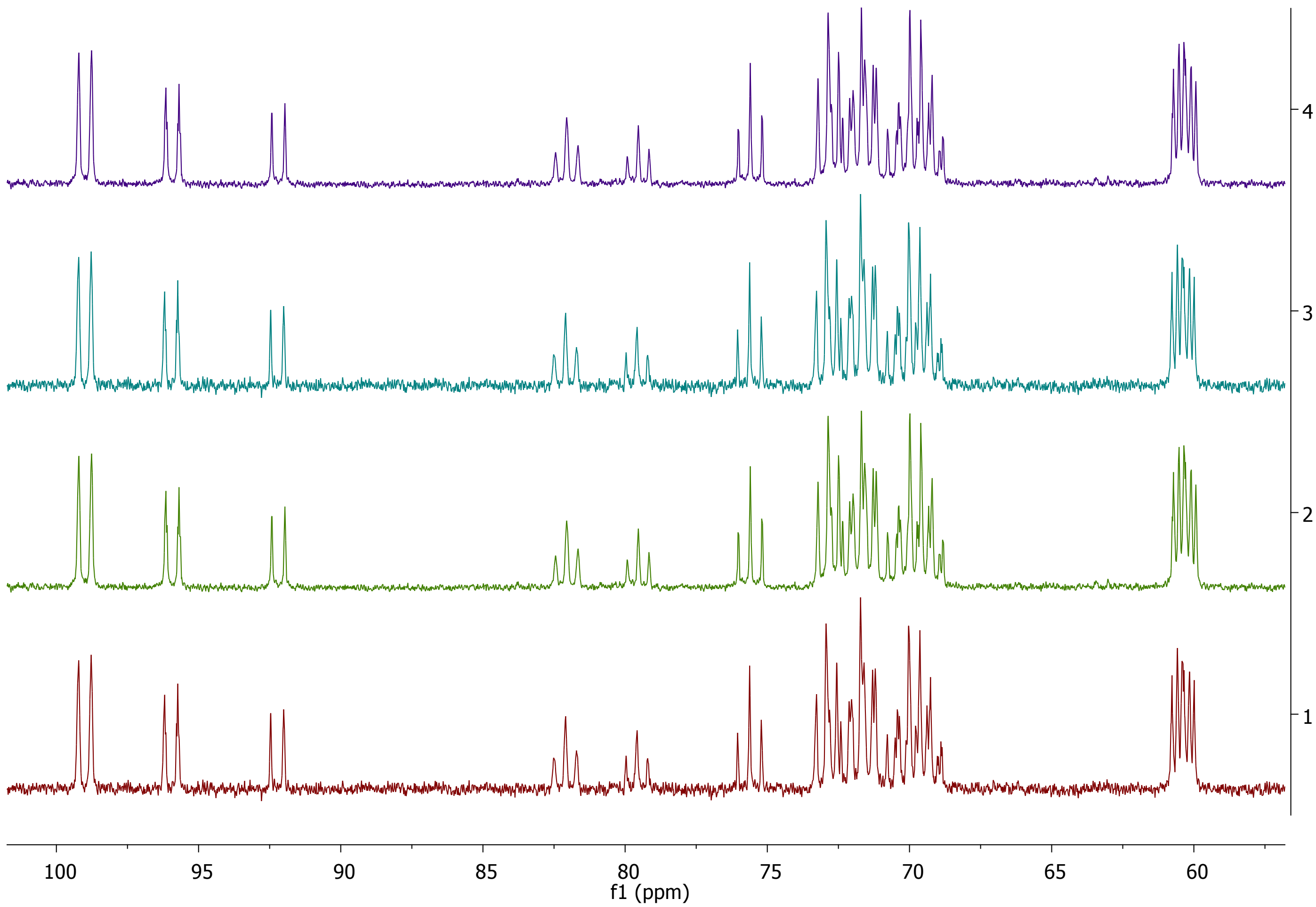

Figure S11. ***In vitro*** **serum stability of [U-^{13}C]sakebiose in human serum without α-glucosidase inhibitor.** The 100 MHz ^{13}C NMR spectrum of [U-^{13}C] sakebiose in D_2O (std) and human serum incubated for 20, 40, and 60 min respectively. The peak intensity of [U-^{13}C] sakebiose was intact over time due to the absence of alpha glucosidase activity.

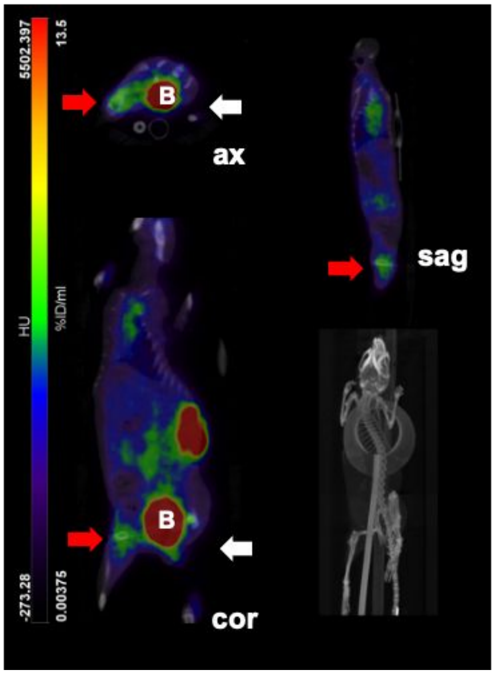

Figure S12. Whole body μPET/CT imaging using [2-^{18}F]maltose (N=3). The site of live inoculation is indicated by a red arrow, control side indicated by a white arrow. Axial (ax), coronal (cor), sagittal (sag).

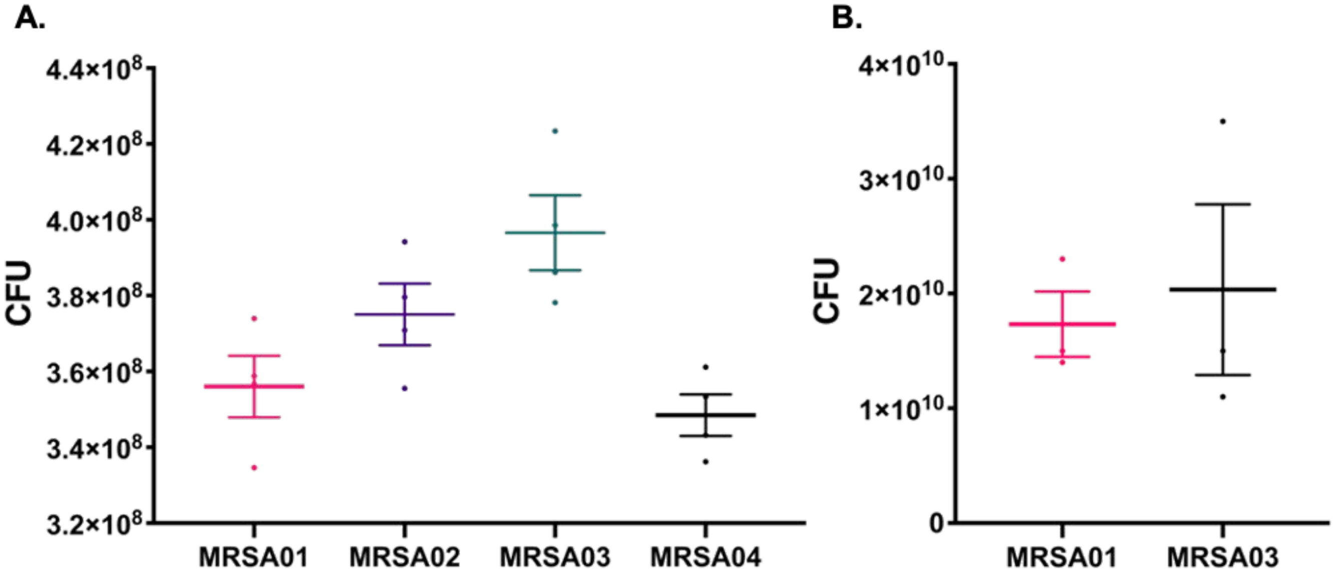

Figure S13. MRSA corresponding colony forming units (CFU). **A.** *In vitro,* MRSA strains 01 to 04 were grown overnight in LB in a shaking incubator at 37 °C. Overnight cultures were diluted to an optical density at 600 nm (OD_{600}) of 0.05 and grown to exponential phase (~ 0.4). Bacterial cultures where then incubated with 1mM of maltose for 2h. After incubation samples were centrifuged and counted by serial dilution and plating on LB agar plates. **B.** *In vivo*, harvested infected muscle was homogenized to determine CFU by serial dilution, plating onto LB agar plates and counting.

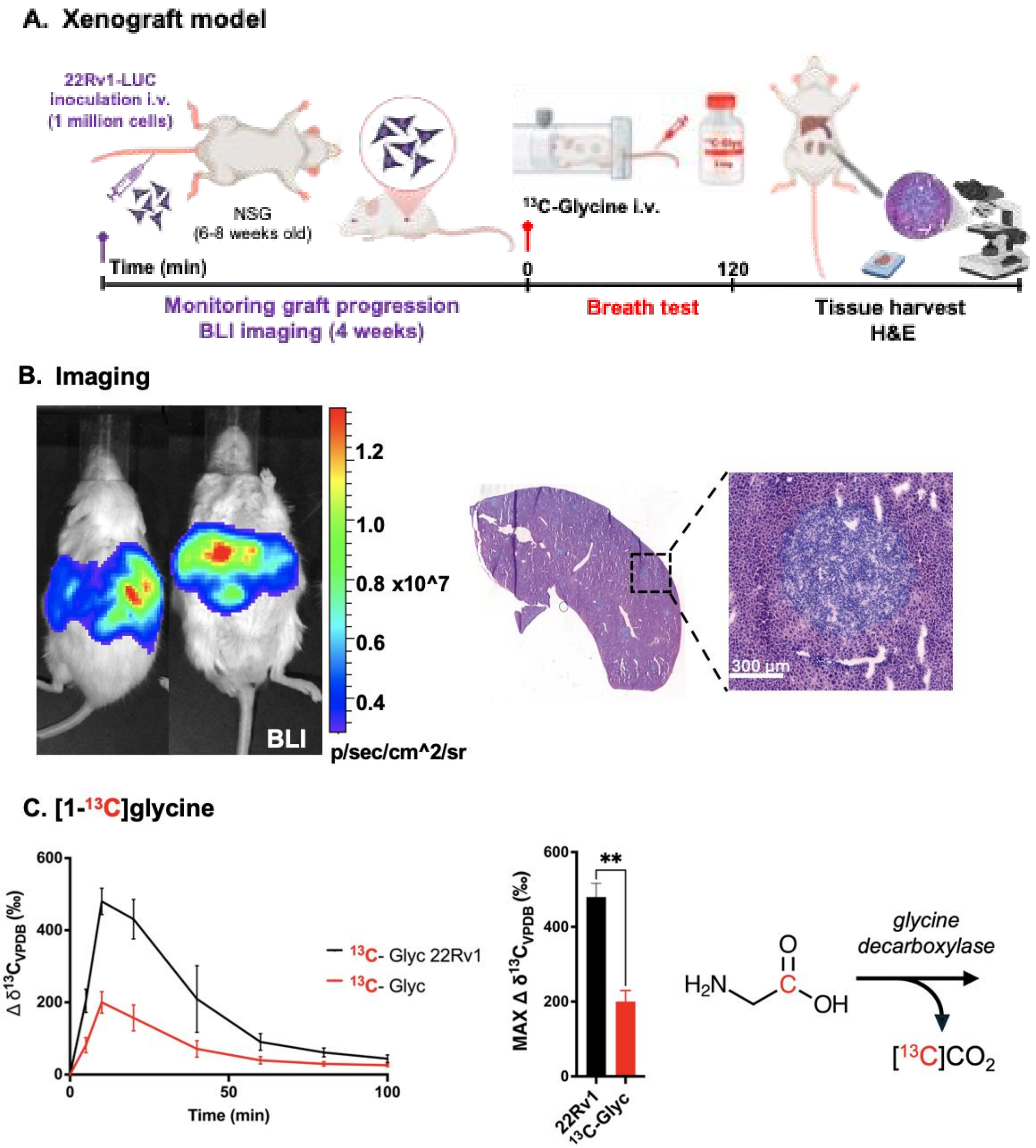

Figure S14. ***In vivo*** **[^{13}C]CO_2 production by 22Rv1 prostate cancer xenografts following intravenous [1-^{13}C]glycine administration. A-B.** Prostate cancer xenograft model. 22Rv1 xenografts were monitored via bioluminescent imaging for approximately 4 weeks followed by intravenous [1-^{13}C]glycine administration and detection of [^{13}C]CO_2 production in a metabolic chamber for 120 minutes. Tumors were analyzed ex vivo via standard immunohistochemistry. **C.** Production of [^{13}C]CO_2 was compared for xenografts (N = 4) versus matched control animals (N = 4) showing increased dynamic signals. The maximum $\Delta\ \delta^{13}C_{VPDB}$ (‰) was 2.4-fold higher in tumor mice (**P-value = 0.0010). This difference was likely due to the established increased expression of glycine decarboxylase (EC 1.4.4.2) in 22Rv1 tumors.

Table S1.

	IRMS*	NDIR**	ICOS***
Instrument cost	High	Low	Med
Consumables cost	High	Med	Low
Precision	High	Low	Med
Speed of analysis	Low	Med	High
[CO_2] concentration	Low	High	Med
Skills required	High	Med	Low

Manufacturers: *Sercon, Elementar, ThermoFisher, Ametek; **Otsuka, Campro Scientific; ***ABB, Picarro, Li-Cor, Aerodyne Research

Table S2. Bacterial strains.
The bacterial strains included in this study are listed in the table below.

Strain	Phenotype or Genotype	Source or Reference
S. aureus (SA)	Wild-type	ATCC 12600
S. aureus Xen36	ATCC 49525 expressing *Photorhabdus luminescens lux ABCDE* operon	Xenogen USA
MRSA 1	Methicillin-resistant *Staphylococcus aureus*	Clinical isolate, University of Nebraska Medical Center
MRSA 2	Methicillin-resistant *Staphylococcus aureus* Clinical isolate	Clinical isolate, University of Nebraska Medical Center
MRSA 3	Methicillin-resistant *Staphylococcus aureus*	Clinical isolate, University of Nebraska Medical Center
MRSA 4	Methicillin-resistant *Staphylococcus aureus*	Clinical isolate, University of Nebraska Medical Center
S. epidermidis (SE)	Wild-type	ATCC 35984
E. coli (EC)	Wild-type	ATCC 25922
S. typhimurium (ST)	Wild-type	ATCC 29630
E. cloacae (ECL)	Wild-type	ATCC 7256
E. faecalis (EF)	Wild-type	ATCC 19433

Table S3. ^{13}C labeled compounds.
The ^{13}C-labeled compounds included in this study are listed in the table below.

Compound	Specifications	Source or Reference
Sorbitol (Sor)	D-[UL-$^{13}C_6$]sorbitol [$^{13}C_6H_{14}O_6$]	OMICRON BIOCHEMICALS INC
Glucose (Glu)	D-[UL-$^{13}C_6$]glucose [$^{13}C_6H_{12}O_6$]	OMICRON BIOCHEMICALS INC
Urea	Urea-^{13}C [$H_2N^{13}CONH_2$]	Sigma-Aldrich
Maltose (Mal)	[UL-$^{13}C_{12}$]maltose monohydrate [$^{13}C_{12}H_{22}O_{11}\cdot H_2O$]	OMICRON BIOCHEMICALS INC
Maltotriose (Mtri)	[UL-$^{13}C_{18}$]maltotriose hydrate [$^{13}C_{18}H_{32}O_{16}\cdot(H_2O)_x$]	OMICRON BIOCHEMICALS INC
Mannitol (Man)	D-[UL-$^{13}C_6$]mannitol [$^{13}C_6H_{14}O_6$]	OMICRON BIOCHEMICALS INC
Xylose (Xyl)	D-[UL-$^{13}C_5$]xylose [$^{13}C_5H_{10}O_5$]	OMICRON BIOCHEMICALS INC
Arabinose (Ara)	L-[UL-$^{13}C_5$]arabinose [$^{13}C_5H_{10}O_5$]	OMICRON BIOCHEMICALS INC

B. Synthetic Procedures:

B1.General:

All chemical reagents were purchased from commercial sources (Acros Organics, Alfa Aesar, AK Scientific, Omicron Biochemicals & Sigma-Aldrich) and used without further purification unless otherwise stated. ^{1}H, ^{13}C, ^{31}P spectra were obtained on a Bruker Avance III HD 400 MHz instrument at the UCSF Nuclear Magnetic Resonance Laboratory and data were processed using MestReNova. Abbreviations are as follows: s (singlet), d (doublet), t (triplet), q (quartet), m (multiplet). High resolution mass spectra (HRMS) services were provided by University of California, Berkeley Spectrometry Facility. Analytical HPLC was performed using a Waters pump equipped with a manual Rheodyne injector (1 mL loop), a refracted index (RI) detector. The stationary phase was YMC-Pack Polyamine II column and the mobile phase of 73:27 acetonitrile/H_2O at a flowrate of 1 mL/min. For semi prep HPLC, a YMC-Pack Polyamine II stationary phase was used with a mobile phase of 73:27 acetonitrile/H_2O at a flowrate of 3 mL/min.

B2. Expression and purification of A1NP (sakebiose phosphorylase)

A1NP was expressed from an inducible vector construct (pET21a) in *E. coli* BL21 (DE3). Precultures were grown overnight in 5 mL lysogeny broth (LB) medium (10 g/L tryptone, 5 g/L yeast extract, 2.5 g/L NaCl) with the appropriate amount of antibiotic (100 µg/mL ampicillin) at 37 °C at 200 rpm. Next, 1% v/v was inoculated in 250 mL fresh LB containing the appropriate antibiotic (i.e. 100 µg/mL ampicillin) and further incubated under the same conditions. When the OD600 reached a value of about 0.6, enzyme expression was induced by adding isopropyl β-D-1-thiogalactopyranoside (IPTG) to a final concentration of 0.1 mM. Cultures were then further incubated overnight at 20 °C and 200 rpm. Hereafter, cells were harvested through centrifugation at 9000 rpm and 4 °C for 30 min using a Sorvall RC 6+ centrifuge (Thermo Scientific). The obtained pellets were stored at -20 °C until further use. For enzyme extraction and purification, cell pellets were thawed and dissolved in 8 mL lysis buffer consisting of 10 mM imidazole, 300 mM NaCl, 0.1 mM phenylmethylsulfonyl fluoride (PMSF), 1 mg/mL lysozyme and 50 mM phosphate buffer; pH 7.4. This suspension was incubated on ice for 30 min and

sonicated 3 times 2 min (Branson sonifier 250, level 3, 50% duty cyle). Finally, the debris was removed by centrifugation (9000 rpm, 1 h). The supernatant was purified by means of His-tag Ni-affinity chromatography. A column containing 1 mL of bed volume HisPur™ Ni-NTA resin (Thermo Fisher) was equilibrated with 6 mL of equilibrium buffer (10 mM imidazole, 50 mM NaH_2PO_4 and 300 mM NaCl; pH 7.4). The enzyme supernatant was added and the column was washed two times with 4 mL of wash buffer (30 mM imidazole, 50 mM NaH_2PO_4 and 300 mM NaCl; pH 7.4). Then, the enzyme was eluted using 8 mL of elution buffer (250 mM imidazole, 50 mM NaH_2PO_4 and 300 mM NaCl; pH 7.4). To wash away the denaturing imidazole and alter the buffer to MES-buffer (50 mM, pH 6.5), the elution fraction was collected in a 50-kDa cutoff Amicon centrifugal filter unit (Merck Millipore) for buffer exchange. The enzymes were then stored at -20 °C in an Eppendorf tube. Protein concentration was measured with a Nanodrop ND-1000 (Thermo Scientific) using the extinction coefficients calculated with the ProtParam tool on the ExPASy server (https://web.expasy.org/protparam/).

B3. Synthesis of [U- ^{13}C]sakebiose

The precursor β-D-Glucose-1-phosphate (βGlc1-P, U-^{13}C) was synthesized as previously described[1]. U-^{13}C -β-D-glucose-1-phosphate (20 mg, 0.07516 mmol) and U-^{13}C-D-glucose (36 mg, 0.1935 mmol) were dissolved in MES buffer (50 mM, pH 6.5, 0.3 mL) in a reaction vial, and sakebiose phosphorylase (**A1NP**) (0.6 mg, lyophilized powder) was added. The mixture was incubated at 37 °C for 6–24 h with gentle stirring. The reaction was quenched by addition of MeCN (0.8 mL), and the precipitated protein was removed by filtration. The filtrate was purified by HPLC using a polyamine II semi-preparative column with 73% MeCN/27% H_2O as the mobile phase to afford U-^{13}C-sakebiose.

After completion of the reaction, the mixture was quenched with MeCN (0.8 mL), and the precipitated protein was removed by centrifugation and filtration (0.45 µm PTFE). The filtrate was subjected to semipreparative HPLC purification (polyamine II; isocratic 73:27 MeCN/H_2O; 3–4 mL min^{-1}; RI detection). Product-containing fractions were pooled, concentrated to remove MeCN, and lyophilized to afford [U-^{13}C] sakebiose, as confirmed by HPLC analysis (Fig.2).

¹³C NMR of U-¹³C-sakebiose in D_2O

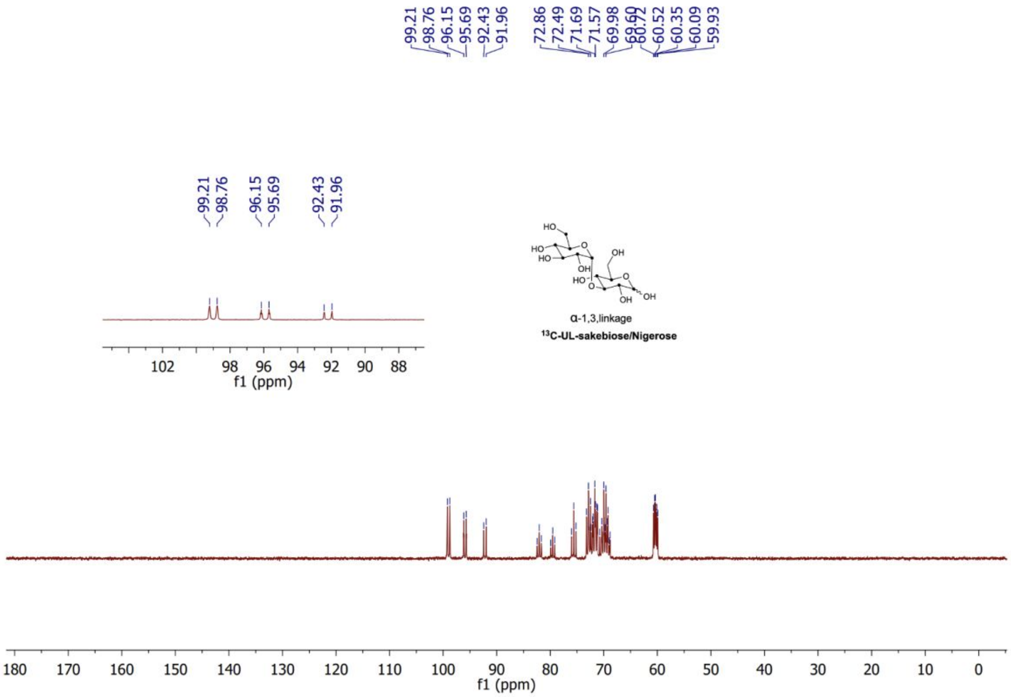

Figure B3.1. NMR Characterization data. ¹³ C NMR (100 MHz, D $_2$ O) δ 98.99 (d, J = 44.9 Hz), 95.92 (d, J = 46.1 Hz), 92.19 (d, J = 45.9 Hz), 82.05 (t, J = 39.7 Hz), 79.54 (t, J = 38.4 Hz), 75.61 (t, J = 41.8 Hz), 73.35 – 72.40 (m), 72.43 – 71.85 (m), 71.82 – 70.62 (m), 70.23 – 68.48 (m), 60.93 – 60.30 (m), 60.42 – 59.78 (m).

¹H NMR of U-¹³C-sakebiose in D_2O

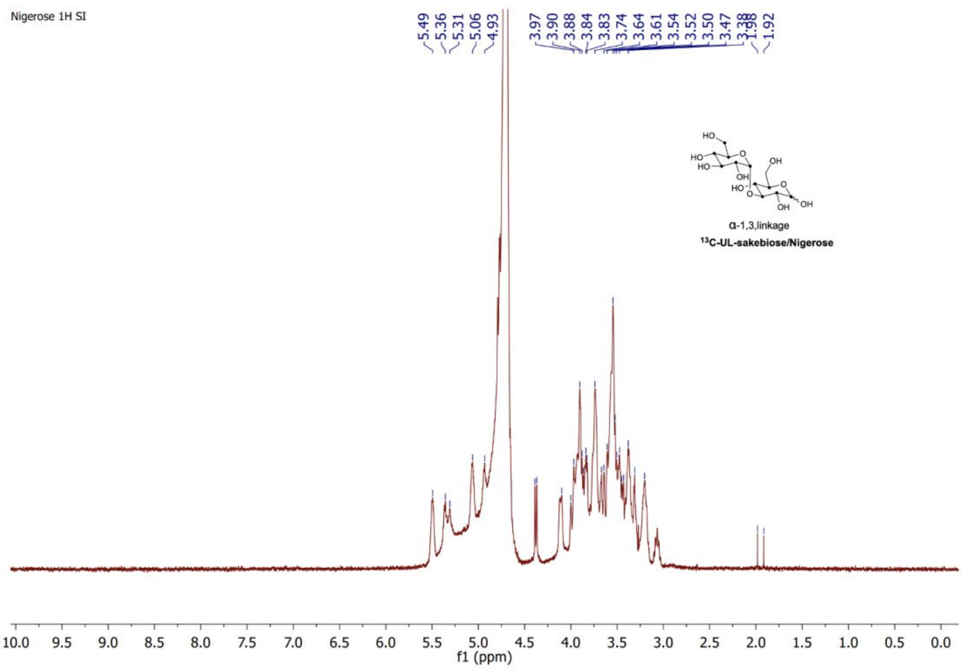

Figure B.3.2. NMR Characterization data. ^{1}**H NMR** (400 MHz, D_2O) δ 5.63 – 5.24 (m, 1H), 5.00 (d, J = 52.8 Hz, 1H), 4.81 (dd, J = 36.8, 29.5 Hz, 2H), 4.89 – 4.57 (m, 2H), 4.51 – 4.05 (m, 1H), 3.88 (m, J = 50.1, 38.8, 23.9 Hz, 3H), 3.72 – 3.29 (m, 3H), 3.26 – 2.94 (m, 1H).

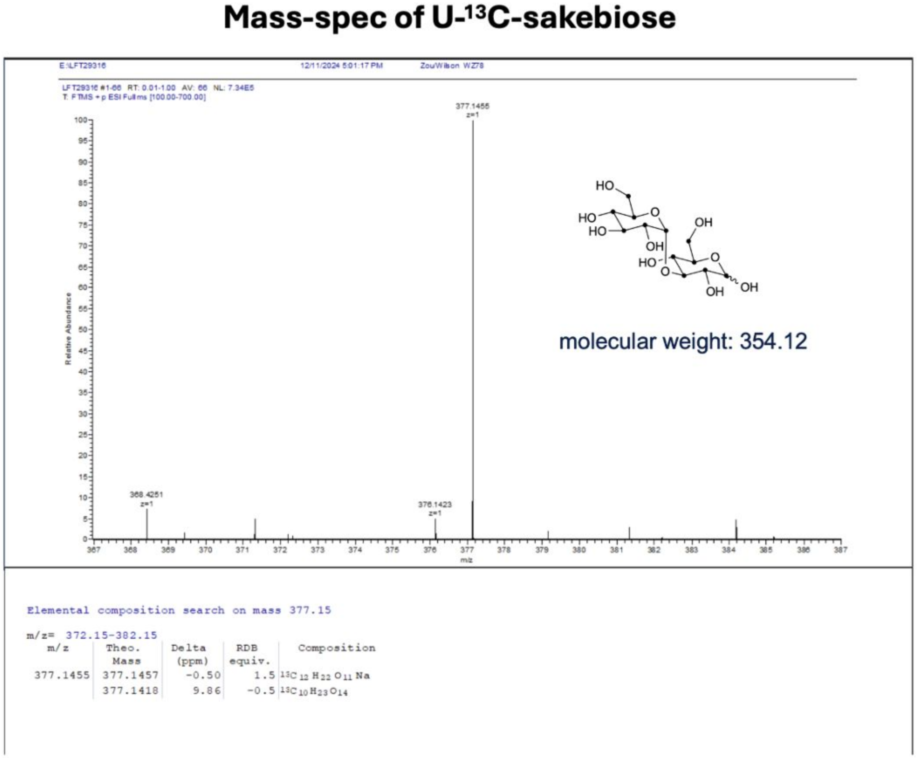

m/z	Theo. Mass	Delta (ppm)	RDB equiv.	Composition
377.1455	377.1457	-0.50	1.5	$^{13}C_{12}H_{22}O_{11}Na$
	377.1418	9.86	-0.5	$^{13}C_{10}H_{23}O_{14}$

Figure B3.3. HRMS (ESI) data U-^{13}C-sakebiose.

C. *In vivo* Procedures

C1. *In vivo* mice models

The mice were housed in individually ventilated cages under normal diet in groups of five throughout the experiment with ad libitum access to food and water. Prior bacteria or tumor injection the mice were anesthetized with 3% isoflurane.

1. **Myositis**

S. aureus and *E. coli* were used to induce a hind limb myositis in 24 CBA/J mice[2]. Both strains were grown in Luria-Bertani (LB) medium overnight at 37 °C and diluted in saline to obtain the desired bacterial load for infection (~10^8 CFU). Bacteria was injected via intramuscular injection. At 12h post infection [U-^{13}C]maltose (2mg, 1mg voglibose /animal), [U-^{13}C]mannitol (2mg/animal) or [U-^{13}C]arabinose (2mg/animal) were

intravenously administered and studied following the above-mentioned protocol. Alternatively, natural abundance maltose (2mg, 1mg voglibose/animal), mannitol (2mg/animal) or arabinose (2mg/animal) was intravenously administered as a control.

Infected tissue of euthanized mice was excised and embedded in optimum cutting temperature (OCT) at -80°C overnight. Afterwards, 10 µm thick sections were cut and stained following Gram staining protocol (American Society for Microbiology (ASM)). Muscle sections were imaged with a Nikon Eclipse Ti microscope (Nikon Instruments Inc.).

Infected tissue was otherwise harvested and homogenized to determine colony forming units (CFU). Homogenized infected muscle was serially diluted and plated onto LB agar plates and incubated at 37 °C overnight followed by counting.

For treatment studies, Ceftriaxone (Sigma) was administered subcutaneously every 90 min (5mg/kg) for 24h.

2. Bacteremia

E. coli was used to induce bacteremia in 16 CBA/J mice. *E. coli* was grown in LB overnight at 37 °C and diluted in saline to obtain the desired bacterial load for infection (~10^6 CFU) and injected via intraperitoneal (IP) injection. At 12h post infection [U-^{13}C]mannitol (2mg/animal) or [U-^{13}C]arabinose (2mg/animal) were intravenously administered and studied following the above-mentioned protocol. Alternatively, natural abundance mannitol or arabinose were intravenously administered as a control.

To determine CFU, liver, kidneys and spleen were excised, homogenized, plated onto LB agar plates and counted by serial dilution and counting.

3. Lung infection

To induce lung infection, *S. aureus* Xen36 (ATCC 49525 with *Photorhabdus luminescens luxABCDE* operon, PerkinElmer, USA) was grown overnight in Todd-Hewitt broth supplemented with 0.2% yeast extract. Bacteria

were grown overnight at 37ºC and diluted in saline to obtain the desire bacteria load for inoculation (~10^7 CFU). C57BL/6J mice (N= 12) were anesthetized with 3% isoflurane (Dechra Pharmaceuticals, England) and inoculated with 100 µL (~10^7 CFU) intranasally[3]. 24h post infection, [U-^{13}C]maltose (2 mg, 1 mg voglibose/ animal) was intravenously administered and studied following the same breath testing protocol. Heat-killed control samples were prepared by incubating the bacterial cultures at 90 °C for 30 min prior intranasal administration. To determine CFU, lungs were excised, homogenized, plated onto LB agar plates and counted by serial dilution and counting.

4. Bone infection

S. aureus Xen36 was grown following the same protocol as for lung inoculations. C57BL/6J mice (N=16) were given 2mg/mL of Metacam (Wedgewood Connect) and 1.3mg/mL of Ethiqa (Fidelis) subcutaneously 20 minutes prior to infection. Mice were anesthetized with 3% isoflurane. Injections were done by plucking the hair from the patella, cleansing the area with aseptic technique, and bone defect created at the distal femur by trephination with a 25-gauge needle. Mice were then inoculated intrafemorally with 2µL of bacteria (~4x 10^7 CFU) delivered through the bone defect into the intramedullary canal. Post infection, mice were given Metacam every 24 hours for 72 hours post infection and supplemental care. Mice femurs were harvested at 7dpi for microCT scans. At days 4 and 8 post infection,^{13}C-enriched maltose was intravenously administered and studied following the same breath testing and CFU protocol.

5. Tumor xenograft

NOD SCID gamma (NSG) mice (N=4) were used to study the baseline metabolism of [1-^{13}C]glycine (3 mg/animal) following the mentioned breath testing protocol. Mice ages 6-8 weeks old were then inoculated intravenously with 1 million 22Rv1-Luc cells in PBS (100 mL). Bioluminescence imaging (BLI) was used to monitor graft progression. After 4 weeks, mice were administered [1-^{13}C]glycine (3 mg/animal) and studied following the same breath testing protocol.

Liver tissue of euthanized mice was excised and embedded in optimum cutting temperature (OCT) at -80°C overnight. Afterwards, 10 µm thick sections were cut and stained with hematoxylin and eosin staining protocol. Tumor sections were imaged with a Nikon Eclipse Ti microscope (Nikon Instruments Inc.)[4].

6. Background metabolism studies

Healthy mice, CBA/J (N= 32), C57BL/6J (N= 4) and NSG (N=4), were used to study the baseline metabolism of the different 13C-enriched metabolites used in the study ([U-^{13}C]maltose (2 mg, 1 mg voglibose/ animal), [U-^{13}C]mannitol (2mg/animal), [U-^{13}C]arabinose (2mg/animal), [U-^{13}C]sorbitol (2mg/animal), [U-^{13}C]glucose (2mg/animal), [^{13}C]urea (2mg/animal), [U-^{13}C]maltotriose (2 mg, 1 mg voglibose/ animal), [U-^{13}C]xylose (2mg/animal) and [1-^{13}C]glycine (3 mg/animal) following the same breath testing protocol.

C2. [^{13}C]CO_2 sample analysis via NDIR

In vitro: After incubation, samples were frozen at -80 °C followed by determinations of $\delta^{13}CO_2$ in the vial headspace. All samples were anonymized (no identifying information) prior to analysis and each vial was measured using a NDIR ^{13}C analyzer (Helifan FanCi; Campro Scientific). Internal $^{13}CO_2$ calibrations were conducted before each round of measurements and vials containing externally validated laboratory standard gases were run in triplicate in series for every N=12 breath samples. The δ^{13}C values are reported in terms of $^{13}C_{VPDB}$ and all measurements were made within four weeks of collection [5–7].

In vivo: After metabolite administration via intravenous (IV) injection the mice were quickly relocated to individual 1-liter metabolic chambers attached to a positive/negative pump (Fristaden Lab) for the duration of the experiment [8]. The metabolic chambers were periodically sealed for 2 minutes to allow the CO_2 levels to increase at 0-, 10-, 20-, 40-, 80-, and 100-minutes post-metabolite injection. A 6 mL subsample of the gas inside each metabolic chamber was then transferred by negative pressure into a previously evacuated 12 mL vial. Baseline breath samples were also collected before ^{13}C-metabolite administration. The breath samples

were frozen at -80 °C until [^{13}C]CO_2 analysis as described above. The breath testing procedure was performed with 4 mice simultaneously at ~37°C.

D. References

1. Sorlin, A. M. *et al.* Chemoenzymatic Syntheses of Fluorine-18-Labeled Disaccharides from [18F] FDG Yield Potent Sensors of Living Bacteria In Vivo. *J Am Chem Soc* **145**, 17632–17642 (2023).
2. Craig, W. A., Redington, J. & Ebert, S. C. Pharmacodynamics of amikacin in vitro and in mouse thigh and lung infections. *J Antimicrob Chemother* **27 Suppl C**, 29–40 (1991).
3. Bergamini, G. *et al.* Mouse pneumonia model by Acinetobacter baumannii multidrug resistant strains: Comparison between intranasal inoculation, intratracheal instillation and oropharyngeal aspiration techniques. *PLoS One* **16**, e0260627 (2021).
4. Bidkar, A. P. *et al.* Effective Treatment of Disseminated Prostate Cancer Using CD46-Targeted 225Ac Therapy. *Clinical Cancer Research* OF1–OF15 (2025) doi:10.1158/1078-0432.CCR-24-2850.
5. Israeli, E., Ilan, Y., Meir, S. B., Buenavida, C. & Goldin, E. A novel 13C-urea breath test device for the diagnosis of Helicobacter pylori infection: continuous online measurements allow for faster test results with high accuracy. *J Clin Gastroenterol* **37**, 139–41 (2003).
6. Shirin, H. *et al.* Evaluation of a novel continuous real time (13)C urea breath analyser for Helicobacter pylori. *Aliment Pharmacol Ther* **15**, 389–94 (2001).
7. Braden, B. *et al.* Clinically feasible stable isotope technique at a reasonable price: analysis of 13CO2/12CO2-abundance in breath samples with a new isotope selective-nondispersive infrared spectrometer. *Z Gastroenterol* **32**, 675–8 (1994).
8. McCue, M. D. CO2 scrubbing, zero gases, Keeling plots, and a mathematical approach to ameliorate the deleterious effects of ambient CO2 during 13 C breath testing in humans and animals. *Rapid Commun Mass Spectrom* **37**, e9639 (2023).
