# Supplementary material for: Detecting Bacteria in Their Mammalian Hosts Using Metabolism-Targeted [13C]CO2 Breath Testing
Source: ACS Cent Sci. 2026 Mar 18;12(4):457–72. doi: 10.1021/acscentsci.5c01995 (PMC13107215; doi:10.1021/acscentsci.5c01995)
Supplement: Supplementary file 2 [file oc5c01995_si_002.pdf]

oc-2025-019955.R1

Name: Peer Review Information for "Detecting bacteria in their mammalian hosts using metabolism-targeted [13C]CO2 breath testing"

First Round of Reviewer Comments

Reviewer: 1

Comments to the Author

This is a very interesting work, the idea of studying microbial metabolism within a host is rather undeveloped compared to other potential infectious disease diagnostics. I have a few comments.

Fig 2 legend is confusing, are you saying S aureus is urease negative? This is not the case.

Fig 6a legend. Pneumoniae would imply a different species. Correct.

Several time course graphs with only color coding are difficult to decipher. Add symbols or other tools to make these clear.

I am not an IUPAC person, but is the use of naming, as in [13C]CO2, correct?

Reviewer: 2

Comments to the Author

This study presents the development of bacteria-specific carbohydrate probes used to detect bacterial infections by [13C]CO2 breath testing. In principle, [13C]CO2 breath testing has been previously explored in the context H. pylori infections which generate

[13C]CO2 from [13C]urea through urease activity. Previous studies have also explored [13C]CO2 breath testing using other 13C labeled substrates like glucose, lactose, phenylalanine, octanoate, and leucine to probe various aspects of substrate utilization. Additionally, breath testing has already had prior success in detecting bacterial-infections through hydrogen and methane detection, allowing for an isotope free assay of bacterial metabolic activity. Here the authors add new capabilities and insights into this area of research by extending the application of 13C[CO2] breath testing to detect bacterial infections with 13C labeled sugars that are readily metabolized by bacteria with minimal background signal from mammalian cells. The investigators test these probes with a variety of bacterial infection models, demonstrating specificity and a wide range of infections that could be detected with this methodology, and validate their findings with conventional PET imaging using a PET analog of one of the 13C probes used for breath testing. However, this work could be significantly improved by addressing the following major and minor comments.

Major Comments:

1. This research integrated chemoenzymatic approaches for the synthesis of [U-13C]sakebiose which was used for in vitro experiments. While the specific use of sakebiose phosphorylase is new in this study, the authors have previously published on a similar strategy using disaccharide phosphorylase to make a radioactive sakebiose derivative. Given the author's previous work, this work would be more impactful if the investigators could perform in vivo experiments with the novel [U-13C]sakebiose probe developed.

2. An important consideration for human studies is the presence of an active immune system that would respond to bacterial infections. Two of the three animal strains used in this study are immunodeficient. From a method development standpoint, immunodeficient mice are a favorable model to allow for a clear detection of microbial metabolism. Based on the C57Bl/6J experiments it's promising that this method would still work in the presence of an immune response, however, it would be important to include a comparative experiment between immunocompetent and immunocompromised mice in the same bacterial infection model to gauge the effects of the immune system on bacterial metabolism. Does an active immune response to infection modulate the transporters or rate limiting enzymatic steps in bacteria during active infection?

3. Majority of clinical breath tests require some form of fasting to control for potential background or non-specific signals. This study does not address the prandial states of the animals examined. Please clarify if animals were in the fed state or fasted state. An important inclusion to this study would be an experiment to ascertain the effects of fasting on bacterial metabolism.

4. While the investigators strictly focused on IV administration given prior PET imaging data, it would be interesting to compare the performance of IV tracer administration against oral tracer administration, especially since differential metabolism has been observed between 18F PET probes and 14C probes which would be metabolized like 13C probes. Additionally, prior work with 13C-phenylalanine breath testing found that IV dosing achieved max 13C[CO2] production within 10 min of tracer administration whereas oral dosing took 30-60 min. This is attributed to the complicated kinetics of gastric resorption of substrate. It would be interesting to know how some of the substrates used in this study would perform with oral dosing. This is important because some bacterial infections like H pylori can infect the gastrointestinal track which, along with other gastrointestinal infections, is also an important type of infection to assess clinically. This study demonstrates minimal 13C[CO2] production when tracer is administered to infection free mice – suggesting that IV dosing in this context is incapable of detecting the metabolism of native microbiota in the gut or other organs. Can oral dosing generate signal?

Minor Comments:

1. Some of the sugars like maltose can cause an insulin response, how does this affect bacterial metabolism in the models used?

2. Line 37 – spell out H. pylori on first instance

3. Line 49 – missing oxford comma

4. Line 50 – metabolite should be plural

5. Line 67 – spell out MRI on first instance

6. Lines 94-98 – citation for 13C-xylose is missing

7. Lines 114-115 – maybe reword infectious diseases to bacterial infections

8. Lines 149-150 – missing commas in list

9. Line 157 – CO2 needs subscript

10. Fig.4B – Is the max plot taken from the time course on the left? It seems that the standard deviations are not matching (Check 5B, 6B, 7C) Fig 5B – missing Max from the y-axis

11. Line 284 – phosphotransferase is misspelled

12. Line 297 – maybe "for the non-invasive detection of ..."

13. Given that the bacteremia model administered bacteria via IP and that blood was not checked for CFUs, is it more appropriate to name the model peritonitis?

14. Please describe the solvent composition of the tracers administered IV for breath studies

Reviewer: 3

Comments to the Author

This is an interesting and novel proposed approach to assessing infection that is based on more than two decades of discoveries in the field of pathogen-specific imaging (which capitalizes on pathogen-specific metabolism). While the manuscript is well written, there

are major inherent limitations for this approach that need to be considered and discussed in more details for this manuscript to be acceptable for publication:

1. While pathogen-specific imaging can usually identify the location of the infection(s), the current approach, if successful, would merely indicate the presence of “bacteria” in the system. The location and/or number of infectious loci cannot be inferred. Even when complemented with other imaging data suggesting localized infectious processes, it would still be an inference rather than documentation or “diagnosis” of bacterial infection.

2. While some of the proposed metabolites do have a certain degree of bacterial specificity, many (especially those tested in vivo such as 18F-maltose) can be metabolized by many bacteria including gram negative and gram-positive species. The investigators mention that the proposed approach “also represents a stand-alone diagnostic tool for non-invasive detecting of bacterial infection”. This is an exaggeration since more often tahn not, the proposed approach would not specify which bacterial species/strain infection that is (without additional invasive procedures) and as a result would not be clinically useful for management or treatment.

3. The authors have addressed some limitations, however additional ones need to be elaborated on. For example, which 13C-metabolites will be used in a certain clinical setting? Or is it a matter of serial testing to narrow the possibilities?

4. Another major potential hurdle that is mentioned only briefly is the metabolite doses that would be needed in humans. In mice, 2-3 milligrams were administered per animal. What would the corresponding human dose (estimated range at least) be in their opinion? This is very important since some of those metabolites have well defined systemic effects that could be dangerous. For example, would 13C-mannitol doses be in the clinical range of mannitol administered for intracranial pressure management? The authors mention that: “Regarding dose, breath testing using [13C]urea for detection of H. pylori has been incredibly sensitive and robust, even when low doses (20-100 mg) are used.” This is a bit misleading: It might be safe to administer large amounts orally however intravenous administration is a whole different ball game. A thorough discussion of the projected dose requirements for human translation is needed as well as the associated pitfalls.

The described approach, however, could potentially be very useful in the setting of an already diagnosed specific infectious process, where breath tests (if successfully validated in humans) can be used for noninvasive follow up of treatment response. As such, this methodology could be complementary to available diagnostic, laboratory and clinical techniques, in view of its simplicity and the commercial availability of the 13C-labeled metabolites (mentioned in the manuscript, not verified by the reviewer). That of course would depend on the sensitivity/ granularity of the measurements. In other words, will the quantification of 13C-CO2 be sensitive enough to detect minor changes in concentrations over time in treated patients? Obviously that can't be predicted a priori however should be discussed as a potential limitation.

Additional comments:

- How did the investigators determine the metabolite doses to be used for in vivo animal testing (2 to 3 milligrams per animal)? in vitro assessment of minimum required doses are described. Were ascending doses similarly tested in animals to determine the minimum required dose for elucidating differences in CO2 levels between infected and uninfected mice?
- doses of 2-3 mg per mouse are used. Metabolite concentrations and volumes used in vivo should be listed in more details.
- Even if the 13C-metabolites are commercially-available, this does not mean they will be equally available in cGMP-compliant form for human use. This should be discussed as a reflection on the feasibility of human translation.

Author's Response to Peer Review Comments:

Response to Critiques:

Reviewers' Comments:

Reviewer # 1:

General comments:

This is a very interesting work, the idea of studying microbial metabolism within a host is rather undeveloped compared to other potential infectious disease diagnostics. I have a few comments.

We appreciate these positive remarks.

R1.1. Fig 2 legend is confusing, are you saying S aureus is urease negative? This is not the case.

Thank you for this correction. As the reviewer states, over 90% of *S. aureus* are urease positive. The strain we studied was urease negative (we have verified using BBL urease test broth). The legend and text have been updated to correct this error.

R1.2. Fig 6a legend. Pneumoniae would imply a different species. Correct.

Thank you, this error has been corrected in the revised manuscript.

R1.3. Several time course graphs with only color coding are difficult to decipher. Add symbols or other tools to make these clear.

Thank you for this comment. This critique is particularly relevant for **Figure 3A**, which has been updated with symbols for the different metabolites.

R1.4. I am not an IUPAC person, but is the use of naming, as in [13C]CO2, correct?

We have reviewed ACS guidelines and believe that for a single-carbon molecule "[^{13}C]CO_2" and "[^{13}C]urea" are correct whereas for a universally ^{13}C-enriched molecule "D-[U-^{13}C]glucose," "[U^{13}C]maltose and analogous are correct. There is some variability in the chemical literature so we would be happy to change this naming scheme at the discretion of *ACS Central Science*. We will also contact the journal editorial staff directly.

Reviewer # 2:

General comments:

This study presents the development of bacteria-specific carbohydrate probes used to detect bacterial infections by [13C]CO2 breath testing. In principle, [13C]CO2 breath testing has been previously explored in the context H. pylori infections which generate [13C]CO2 from [13C]urea through urease activity. Previous studies have also explored [13C]CO2 breath testing using other 13C labeled substrates like glucose, lactose, phenylalanine, octanoate, and leucine to probe various aspects of substrate utilization.

Additionally, breath testing has already had prior success in detecting bacterial-infections through hydrogen and methane detection, allowing for an isotope free assay of bacterial metabolic activity. Here the authors add new capabilities and insights into this area of research by extending the application of 13C[CO2] breath testing to detect bacterial infections with 13C labeled sugars that are readily metabolized by bacteria with minimal background signal from mammalian cells. The investigators test these probes with a variety of bacterial infection

models, demonstrating specificity and a wide range of infections that could be detected with this methodology, and validate their findings with conventional PET imaging using a PET analog of one of the 13C probes used for breath testing. However, this work could be significantly improved by addressing the following major and minor comments.

We greatly appreciate the reviewer's interest in the manuscript and breath testing field.

R2.1. This research integrated chemoenzymatic approaches for the synthesis of [U13C]sakebiose which was used for in vitro experiments. While the specific use of sakebiose phosphorylase is new in this study, the authors have previously published on a similar strategy using disaccharide phosphorylase to make a radioactive sakebiose derivative. Given the author's previous work, this work would be more impactful if the investigators could perform in vivo experiments with the novel [U-13C]sakebiose probe developed.

Thank you for this comment. We agree that since the synthesis and *in vitro* testing of [U^{13}C]sakebiose was originally presented early in the manuscript, it appeared appropriate to test this compound *in vivo* and potentially in advanced preclinical models of infection. At present, the chemoenzymatic synthesis of [U-^{13}C]sakebiose is not optimized and yields insufficient ^{13}Cenriched compound for *in vivo* studies. The synthesis of [U-^{13}C]sakebiose was performed via reverse phosphorolysis via sakebiose phosphorylase using [illegible]-D-[U-^{13}C]glucose-1-phosphate as a precursor[1]. At the time of this writing, the natural abundance version of this molecule sells for ~ $500/ 5 mg at Sigma Aldrich. Therefore, future studies are planned using chemoenzymatic radiosyntheses more amenable to scale-up, likely generating a partially ^{13}C-enriched sakebiose. Prof. Desmet's lab (author of the current manuscript) used a sucrose phosphorylase variant (L341I_Q345S) to synthesize > 3 kg of the "rare" disaccharide kojibiose[2]. We will adapt a similar strategy to make a > 50% ^{13}C-enriched sakebiose, but this is a topic for a future manuscript focusing on scale-up for patient studies, and other ^{13}C-enriched disaccharides. The other substrates studied in the current paper are universally ^{13}C-enriched leading us to use the sakebiose phosphorylase/ reverse phosphorolysis method to synthesize [U-^{13}C]sakebiose. This reviewer comment has been addressed in the revised manuscript in two ways:

1. The importance of chemoenzymatic scale-up has been more fully discussed, in particular the sucrose-phosphorylase strategy mentioned above.
2. The synthesis of [U-^{13}C]sakebiose and its *in vitro* testing are moved to the end of the manuscript to highlight a promising new direction for bacteria-specific [^{13}C]CO_2 breath testing and the basis for future work.

R2.2. An important consideration for human studies is the presence of an active immune system that would respond to bacterial infections. Two of the three animal strains used in this study are immunodeficient. From a method

development standpoint, immunodeficient mice are a favorable model to allow for a clear detection of microbial metabolism. Based on the C57Bl/6J experiments it's promising that this method would still work in the presence of an immune response, however, it would be important to include a comparative experiment between immunocompetent and immunocompromised mice in the same bacterial infection model to gauge the effects of the immune system on bacterial metabolism. Does an active immune response to infection modulate the transporters or rate limiting enzymatic steps in bacteria during active infection?

Thank you for this important comment. A key feature of pathogen-targeted diagnostics is their ability to differentiate infection from sterile inflammation, which is why immunocompetent mice are typically used[3,4]. As the reviewer suggests, an active immune response to infection can modulate bacterial transporters and rate-limiting enzymatic steps during active infection. The two animal strains used for bacterial experiments are immunocompetent (CBA/J, C57Bl/6J). The third strain included in the manuscript, NOD SCID gamma (NSG), is only used for the described tumor xenograft model for which immunodeficient mice are necessary.

R2.3. Majority of clinical breath tests require some form of fasting to control for potential background or non-specific signals. This study does not address the prandial states of the animals examined. Please clarify if animals were in the fed state or fasted state. An important inclusion to this study would be an experiment to ascertain the effects of fasting on bacterial metabolism.

This is an important comment. In the original manuscript, the animals studied were in the fed state. A typical [^{18}F]FDG-PET scan (representing ~ 80% of PET scans performed in the United States) usually requires fasting for 4-6 hours before the study[5]. The effect of host fasting on pathogen-targeted metabolite incorporation is poorly understood and to our knowledge not previously reported. The manuscript has been revised to indicate that the experiments reported in the main text were performed in the fed state. We have reported an additional experiment in the Supplemental Data for a study performed in the fasted state (new **Supp. Fig. S6**). This study using D-[U-^{13}C]mannitol was performed in the in the bacterial *E. coli* myositis model after a 12 hour fast, yielding similar [^{13}C]CO_2 production. Additional studies (in both animal models and humans) will be needed to confirm potential advantages of [^{13}C]CO_2 breath testing in the fasted state, which could affect metabolite uptake by pathogens.

R2.4. While the investigators strictly focused on IV administration given prior PET imaging data, it would be interesting to compare the performance of IV tracer administration against oral tracer administration, especially since differential metabolism has been observed between 18F PET probes and 14C probes which would be metabolized like 13C probes. Additionally, prior work with 13C-phenylalanine breath testing found that IV dosing achieved max 13C[CO2]

production within 10 min of tracer administration whereas oral dosing took 30-60 min. This is attributed to the complicated kinetics of gastric resorption of substrate. It would be interesting to know how some of the substrates used in this study would perform with oral dosing. This is important because some bacterial infections like H pylori can infect the gastrointestinal track which, along with other gastrointestinal infections, is also an important type of infection to assess clinically. This study demonstrates minimal 13C[CO2] production when tracer is administered to infection free mice – suggesting that IV dosing in this context is incapable of detecting the metabolism of native microbiota in the gut or other organs. Can oral dosing generate signal?

Thank you for this critique. A key hypothesis of this work is that ^{13}C-enriched metabolites administered intravenously will not detect organisms in the normal mammalian microbiome (mice or humans). This approach contrasts with existing clinical [^{13}C]CO_2 breath testing using orallyadministered [^{13}C]urea detects organisms within the gastrointestinal tract, for example *H. pylori*[6].

Since we are developing general tools to detect bacterial infection, we studied ^{13}C-enriched, intravenously administered metabolites analogous to those used for PET imaging. Based on this reviewer comment, we studied orally administered D-[U-^{13}C]mannitol since it had high sensitivity to *E. coli*, which is present in the normal gastrointestinal tract of mice and humans. We hypothesized that orally administered D-[U-^{13}C]mannitol would show [^{13}C]CO_2 production in normal mice, and in fact observed significant signal (new **Supp. Fig. S5**) representing background that would render the detection of pathogen-produced [^{13}C]CO_2 difficult. As the reviewer predicted, [^{13}C]CO_2 production in normal mice following oral D-[U-^{13}C]mannitol was high and followed a different time course. We hope that these new data are helpful for future breath testing strategies and greatly appreciate the reviewer's suggestion.

R2.5. Some of the sugars like maltose can cause an insulin response, how does this affect bacterial metabolism in the models used?

Thank you for this thoughtful comment. We agree that systemically administered sugars can trigger an insulin response, which in turn may influence host glucose metabolism. Although bacteria don't have insulin receptors, both sugar metabolism and host insulin response could influence background signals. As reported in our study, we did not observe background signals in normal mice following the intravenous administration of several ^{13}C-enriched metabolites. The next step will be testing these metabolites in normal humans to exclude the absence of significant background before studying clinical infections.

R2.6. Line 37 – spell out H. pylori on first instance

This error has been corrected in the revised manuscript.

R2.7. Line 49 – missing oxford comma

This error has been corrected in the revised manuscript.

R2.8. Line 50 – metabolite should be plural

This error has been corrected in the revised manuscript.

R2.9. Line 67 – spell out MRI on first instance

Thank you for this correction. Magnetic resonance imaging (MRI) has been updated.

R2.10. Lines 94-98 – citation for 13C-xylose is missing

Thank you for this comment. The bibliography has been updated to include:

Dellert, S F et al. "The 13C-xylose breath test for the diagnosis of small bowel bacterial overgrowth in children." *Journal of pediatric gastroenterology and nutrition* vol. 25,2 (1997): 153-8.

R2.11. Lines 114-115 – maybe reword infectious diseases to bacterial infections

This error has been corrected in the revised manuscript.

R2.12. Lines 149-150 – missing commas in list

This error has been corrected in the revised manuscript.

R2.13. Line 157 – CO2 needs subscript

This error has been corrected in the revised manuscript.

R2.14. Fig.4B – Is the max plot taken from the time course on the left? It seems that the standard deviations are not matching (Check 5B, 6B, 7C) Fig 5B – missing Max from the yaxis.

This error has been corrected in the revised manuscript.

R2.15. Line 284 – phosphotransferase is misspelled

This error has been corrected in the revised manuscript.

R2.16. Line 297 – maybe "for the non-invasive detection of …"

Thank you for this suggestion; this text has been updated.

R2.17. Given that the bacteremia model administered bacteria via IP and that blood was not checked for CFUs, is it more appropriate to name the model peritonitis?

We appreciate this comment and have discussed it with the infectious disease physicians included on the manuscript. Because the model results in disseminated infection affecting numerous organs, the term “bacteremia” is probably more appropriate than “peritonitis,” which implies focal involvement of the peritoneum. The presence of bacteria in the blood was documented via dilution and plating, but because this was non-quantitative these data were not reported in the final manuscript.

R2.18. Please describe the solvent composition of the tracers administered IV for breath studies

All the tracers were prepared in saline. The Materials and Methods section has been updated to clarify the solvent composition.

Reviewer # 3:

General comments:

This is an interesting and novel proposed approach to assessing infection that is based on more than two decades of discoveries in the field of pathogen-specific imaging (which capitalizes on pathogen-specific metabolism). While the manuscript is well written, there are major inherent limitations for this approach that need to be considered and discussed in more details for this manuscript to be acceptable for publication:

We appreciate these positive comments.

R3.1. While pathogen-specific imaging can usually identify the location of the infection(s), the current approach, if successful, would merely indicate the presence of “bacteria” in the system. The location and/or number of infectious loci cannot be inferred. Even when complemented with other imaging data suggesting localized infectious processes, it would still be an inference rather than documentation or “diagnosis” of bacterial infection.

We agree with this comment and have modified the manuscript accordingly. Any approach based on $[^{13}C]CO_2$ breath testing would not allow localization of bacteria, that requires interpretation of patient symptoms, lab testing, routine imaging (i.e. CT/MRI/ultrasound) and potentially pathogenspecific (or immune-targeted) PET imaging. Culture and sensitivity, requiring tissue and/or biofluid sampling will probably remain the gold standard for precise identification of microorganisms as well as their susceptibility to antibiotics. We anticipate that new diagnostic methods will be used most efficiently (1) in the acute care setting, for which antibiotic management is dominated by empiric therapy and (2) in the context of known infection, to document successful management and/or resolution. Edits to the manuscript reflect the potential use of pathogen-specific $[^{13}C]CO_2$ breath testing to complement other diagnostic methods in clinical practice. Compared to other diagnostic

methods, pathogen-specific [^{13}C]CO_2 breath testing using newer analyzers may be particularly fast and cost-efficient.

R3.2. While some of the proposed metabolites do have a certain degree of bacterial specificity, many (especially those tested in vivo such as 18F-maltose) can be metabolized by many bacteria including gram negative and gram-positive species. The investigators mention that the proposed approach "also represents a stand-alone diagnostic tool for non-invasive detecting of bacterial infection". This is an exaggeration since more often than not, the proposed approach would not specify which bacterial species/strain infection that is (without additional invasive procedures) and as a result would not be clinically useful for management or treatment.

Thank you for these helpful comments. We have removed the assertion that [^{13}C]CO_2 breath testing represents a "stand-alone diagnostic tool" since as the reviewer suggests its results (1) should be interpreted in the context of other diagnostic data and (2) do not offer specific information about the type/strain of bacteria present. The reviewer's comments highlight two distinct goals in detecting microorganisms non-invasively. The first goal would be detection of *all* or *most* bacteria to indicate that likely has a bacterial infection, versus viral infection, rheumatologic or neoplastic disease. The second goal is identification of the specific type of bacteria causing the infection, to direct appropriate antibiotic therapy. For the [^{13}C]CO_2 breath testing approach, as for [2-^{18}F]maltose and other PET imaging methods, the clinical question is probably "Is bacterial infection present?" Therefore, uptake by many bacteria including gramnegative and gram-positive bacteria is potentially a strength, if the metabolites studied are specific for bacterial versus mammalian metabolism. For more information re: the type of bacteria present, combinations of metabolic probes might be used[7], duplex PCR[8], or routine culture/ sensitivity analysis. As above we have modified the manuscript to indicate that the proposed methods are not intended to replace time-consuming but precise tools involving bacterial sampling and culture.

R3.3. The authors have addressed some limitations, however additional ones need to be elaborated on. For example, which 13C-metabolites will be used in a certain clinical setting? Or is it a matter of serial testing to narrow the possibilities?

Thank you for this important comment. The next steps are to test promising ^{13}C-enriched metabolites in uninfected control subjects and clinical infections, to establish robust [^{13}C]CO_2 production. These studies will help us to understand which ^{13}C-enriched metabolites are best for various infections. We do not know at present whether certain ^{13}C-enriched metabolites will show too much background in normal human subjects, or which will be most sensitive for gram-positive or gram-negative infections in man. For the current manuscript, we have proposed that (1) ^{13}Cenriched metabolites will be used in humans

depending on their organism sensitivity and (2) following known infections to resolution will be a powerful application of this technique. In the acute setting, the use of a specific ^{13}C-enriched metabolite might follow the likelihood of certain causative organisms. Vertebral discitis-osteomyelitis[9] is most frequently caused by staphylococcal species and other gram-positive bacteria, so our data supports the use of [$U^{13}C$]maltose when this infection is suspected. Similarly, infections of the kidneys and hepatobiliary system[10,11] are frequently caused by gram-negative species so D-[U-^{13}C]mannitol would be used. The manuscript has been modified to suggest these potential applications.

R3.4. Another major potential hurdle that is mentioned only briefly is the metabolite doses that would be needed in humans. In mice, 2-3 milligrams were administered per animal. What would the corresponding human dose (estimated range at least) be in their opinion? This is very important since some of those metabolites have well defined systemic effects that could be dangerous. For example, would 13C-mannitol doses be in the clinical range of mannitol administered for intracranial pressure management? The authors mention that: "Regarding dose, breath testing using [13C]urea for detection of H. pylori has been incredibly sensitive and robust, even when low doses (20-100 mg) are used." This is a bit misleading: It might be safe to administer large amounts orally however intravenous administration is a whole different ball game. A thorough discussion of the projected dose requirements for human translation is needed as well as the associated pitfalls.

Thank you for these important comments. The revised manuscript has included both an analysis of acceptable doses and the need for careful dose escalation studies in humans. The lack of toxicity for the proposed ^{13}C-enriched metabolites is a major advantage of the proposed method. A 2 mg dose of D-[U-^{13}C]mannitol administered to a 20-gram mouse represents 100 mg/kg. Based on a frequently used allometric scaling method (that uses surface area)[12], the corresponding human dose would be 100/ 12.3 or ~ 8 mg/ kg. The typical dose of intravenous mannitol for elevated intracranial pressure is 250 mg/kg to 1000 mg/kg[13]. Similarly, maltose has been administered intravenously up to 250 mg/kg[14]. Urea has also been given intravenously for treatment of hyponatremia at high doses, with a recent study reporting up to 500 mg/kg[15]. Despite these high doses, two important considerations for [^{13}C]CO_2 breath testing include (1) ideally no physiologic response should be provoked (i.e. the osmotic shifts that might accompany high doses of mannitol) and (2) the dose given should have a reasonable cost. Therefore, studies in patients will be required to determine the minimum doses of ^{13}C-enriched metabolites required for detecting bacterial infection. We have highlighted the need for improved synthetic/ formulation methods (including the need for cGMP materials; as below) in the revised manuscript.

R3.5 The described approach, however, could potentially be very useful in the setting of an already diagnosed specific infectious process, where breath tests (if successfully validated in humans) can be used for noninvasive follow up of treatment response. As such, this methodology could be complementary to available diagnostic, laboratory and clinical techniques, in view of its simplicity and the commercial availability of the 13Clabeled metabolites (mentioned in the manuscript, not verified by the reviewer). That of course would depend on the sensitivity/ granularity of the measurements. In other words, will the quantification of 13C-CO2 be sensitive enough to detect minor changes in concentrations over time in treated patients? Obviously that can't be predicted a priori however should be discussed as a potential limitation.

These comments are very important, and the reason human studies should be prioritized in the near term. Human studies are needed to both ensure limited $[^{13}C]CO_2$ background signals in healthy subjects and show that $[^{13}C]CO_2$ production is possible for infected patients. We have stipulated that the latter can be achieved with small doses given the detection $[^{13}C]CO_2$ in existing $[^{13}C]$urea-based breath testing. We entirely agree that sensitivity is difficult to predict in the proposed clinical applications.

R3.6 How did the investigators determine the metabolite doses to be used for in vivo animal testing (2 to 3 milligrams per animal)? in vitro assessment of minimum required doses are described. Were ascending doses similarly tested in animals to determine the minimum required dose for elucidating differences in CO2 levels between infected and uninfected mice?

These doses were chosen based on (1) the analysis above, namely that this dose (based on a commonly used conversion method between animals and humans) was much (> 20-fold) lower (mg/kg) versus patient reports and (2) minimizing enzyme inhibition, cytotoxic effects and insulin response and (3) the cost of commercially available ^{13}C-enriched metabolites. Ascending doses were not tested as data in humans are considered more relevant and can be evaluated in the near term given low toxicity of the common metabolites studied.

R3.7 Doses of 2-3 mg per mouse are used. Metabolite concentrations and volumes used in vivo should be listed in more details.

Thank you for noticing this. As above (see **Reviewer #2; comment 2.18**) the manuscript has been edited to provide specifics re: IV administrations.

R3.8. Even if the 13C-metabolites are commercially-available, this does not mean they will be equally available in cGMP-compliant form for human use. This should be discussed as a reflection on the feasibility of human translation.

We entirely agree with this comment. In the near term the corresponding authors (Wilson;

Neumann) have access to cGMP-compliant materials and have already prepared cGMP D-[U^{13}C]mannitol at St. Jude for testing in human patients. We anticipate that the cost of cGMPcompliant 13C-enriched materials will decrease significantly once the demand for them, and supplier interest increases. In the hyperpolarized ^{13}C-MRI field, the cost of various ^{13}C-enriched, cGMP metabolites ([1-^{13}C]pyruvic acid, [2-^{13}C]pyruvic acid and others) has decreased significantly (> 50%) over the last several years reflecting the feasibility of human studies. The potential limitation of cGMP costs has been added to the revised manuscript.

REFERENCES:

1. Sorlin, A. M. *et al.* Chemoenzymatic Syntheses of Fluorine-18-Labeled Disaccharides from [18F] FDG Yield Potent Sensors of Living Bacteria In Vivo. *J. Am. Chem. Soc.* **145**, 17632–17642 (2023).
2. Beerens, K. *et al.* Biocatalytic Synthesis of the Rare Sugar Kojibiose: Process Scale-Up and Application Testing. *J. Agric. Food Chem.* **65**, 6030–6041 (2017).
3. Weinstein, E. A. *et al.* Imaging Enterobacteriaceae infection in vivo with 18Ffluorodeoxysorbitol positron emission tomography. *Sci. Transl. Med.* **6**, 259ra146 (2014).
4. Sarkar, S. & Heise, M. T. Mouse models as resources for studying infectious diseases. *Clin. Ther.* **41**, 1912–1922 (2019).
5. Boellaard, R. *et al.* FDG PET/CT: EANM procedure guidelines for tumour imaging: version 2.0. *Eur. J. Nucl. Med. Mol. Imaging* **42**, 328–354 (2015).
6. Mounsey, A. & Leonard, E. A. Noninvasive Diagnostic Tests for Helicobacter pylori Infection. *Am. Fam. Physician* **100**, 16–17 (2019).
7. Parker, M. F. L. *et al.* Evaluating the Performance of Pathogen-Targeted Positron Emission Tomography Radiotracers in a Rat Model of Vertebral Discitis-Osteomyelitis. *J. Infect. Dis.* **228**, S281–S290 (2023).
8. Yang, S. & Rothman, R. E. PCR-based diagnostics for infectious diseases: uses, limitations, and future applications in acute-care settings. *Lancet Infect. Dis.* **4**, 337–348 (2004).
9. Zou, X., Li, X., He, K., Song, Q. & Yin, R. Current knowledge of vertebral osteomyelitis: a review. *Eur. J. Clin. Microbiol. Infect. Dis.* **44**, 213–231 (2025).
10. Acute pyelonephritis in adults. *N. Engl. J. Med.* **378**, 1069 (2018).
11. Cozma, M.-A. *et al.* Acute cholangitis: a state-of-the-art review. *Ann Med Surg (Lond)* **86**, 4560–4574 (2024).
12. Nair, A. B. & Jacob, S. A simple practice guide for dose conversion between animals and human. *J. Basic Clin. Pharm.* **7**, 27–31 (2016).
13. Marshall, L. F., SMith, R. W., Rauscher, L. A. & Shapiro, H. M. Mannitol dose requirements in brain-injured patients. *J. Neurosurg.* **48**, 169–172 (1978).

14. Förster, H., Hoos, I. & Boecker, S. [Tests with human volunteers on parenteral utilization of maltose]. *Z. Ernahrungswiss.* **15**, 284–293 (1976).
15. Decaux, G. *et al.* Actual therapeutic indication of an old drug: urea for treatment of severely symptomatic and mild chronic hyponatremia related to SIADH. *J. Clin. Med.* **3**, 1043–1049 (2014).

oc-2025-019955.R2

Name: Peer Review Information for "Detecting bacteria in their mammalian hosts using metabolism-targeted [13C]CO2 breath testing"

Second Round of Reviewer Comments

Reviewer: 2

Comments to the Author

The authors have addressed all of my previous suggestions.

Reviewer: 3

Comments to the Author

The authors have responded to all this reviewer's comments satisfactorily.

This is a promising venue of research for identification and follow-up of bacterial infections. Human validation is however necessary at this point.

Author's Response to Peer Review Comments:

Thank you so much for these positive critiques and we are very enthusiastic to publish our work in ACS Central Science. We have made the following minor modifications to the manuscript:

1. As requested we have provided a Table of Contents figure at the end of the manuscript.
2. We have added additional institutional grant support and the use of BioRender to the Acknowledgements section.
3. Figure 1 has been modified slightly to remove elements that likely required attribution (and include elements made via BioRender). We apologize for not noticing this previously.

Please do not hesitate to reach out with any questions or concerns.

Sincerely, David Wilson
